# Excitatory neurons and oligodendrocyte precursor cells are vulnerable to focal cortical dysplasia type IIIa as suggested by single‐nucleus multiomics

**DOI:** 10.1002/ctm2.70072

**Published:** 2024-10-23

**Authors:** Yingying Liu, Yinchao Li, Yaqian Zhang, Yubao Fang, Lei Lei, Jiabin Yu, Hongping Tan, Lisen Sui, Qiang Guo, Liemin Zhou

**Affiliations:** ^1^ Department of Neurology The Seventh Affiliated Hospital Sun Yat‐sen University Shenzhen Guangdong China; ^2^ Department of Neurology Third Affiliated Hospital Sun Yat‐sen University Guangzhou Guangdong China; ^3^ Epilepsy Center Guangdong Sanjiu Brain Hospital Guangzhou Guangdong China; ^4^ Department of Epilepsy Center The Second Affiliated Hospital Guangzhou University of Chinese Medicine Guangzhou Guangdong China

**Keywords:** excitatory neurons, focal cortical dysplasia type IIIa, multiomics, oligodendrocyte precursor cells, single‐nucleus sequencing

## Abstract

**Background:**

Focal cortical dysplasia (FCD) is a heterogeneous group of cortical developmental malformations that constitute a common cause of medically intractable epilepsy. FCD type IIIa (FCD IIIa) refers to temporal neocortex alterations in architectural organisation or cytoarchitectural composition in the immediate vicinity of hippocampal sclerosis. Slight alterations in the temporal neocortex of FCD IIIa patients pose a challenge for the preoperative diagnosis and definition of the resection range.

**Methods:**

We have performed multimodal integration of single‐nucleus RNA sequencing and single‐nucleus assay for transposase‐accessible chromatin sequencing in the epileptogenic cortex of four patients with FCD IIIa, and three relatively normal temporal neocortex were chosen as controls.

**Results:**

Our study revealed that the most significant dysregulation occurred in excitatory neurons (ENs) and oligodendrocyte precursor cells (OPCs) in the epileptogenic cortex of FCD IIIa patients. In ENs, we constructed a transcription factor (TF)‐hub gene regulatory network and found *DAB1* ^high^ ENs subpopulation mediates neuronal immunity characteristically in FCD IIIa. Western blotting and immunofluorescence were used to validate the changes in protein expression levels caused by some of the key genes. The OPCs were activated and exhibited aberrant phenotypes in FCD IIIa, and TFs regulating reconstructed pseudotime trajectory were identified. Finally, our results revealed aberrant intercellular communication between ENs and OPCs in FCD IIIa patients.

**Conclusions:**

Our study revealed significant and intricate alterations in the transcriptomes and epigenomes in ENs and OPCs of FCD IIIa patients, shedding light on their cell type‐specific regulation and potential pathogenic involvement in this disorder. This work will help evaluate the pathogenesis of cortical dysplasia and epilepsy and explore potential therapeutic targets.

**Key points:**

- Paired snRNA‐seq and snATAC‐seq data were intergrated and analysed to identify crucial subpopulations of ENs and OPCs in the epileptogenic cortex of FCD IIIa patients and explore their possible pathogenic role in the disease.
- A TF‐hub gene regulatory network was constructed in ENs, and the DAB1high Ex‐1 mediated neuronal immunity was characterstically in FCD IIIa patients.
- The OPCs were activated and exhibited aberrant phenotypes in FCD IIIa patients, and TFs regulating reconstructed pseudotime traectory were identified.
- Aberrant intercelluar communications between ENs and OPCs in FCD IIIa patients were identified.

## Graphical abstract

The most significant dysregulation occurred in excitatory neurons and oligodendrocyte precursor cells in the epileptogenic cortex of FCD IIIa patients.

The *DAB1* ^high^ excitatory neurons subpopulation mediates neuronal immunity characteristically in FCD IIIa.

Western blotting and immunofluorescence validated the changes in protein expression levels caused by some of the key genes.

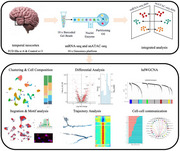

## 1 BACKGROUND

Focal cortical dysplasia (FCD) is a heterogeneous group of malformations of cortical development (MCD) that constitute a common cause of medically intractable epilepsy.^1^ FCD is characterized by abnormal cortical lamination, cell morphology, cellular polarity, and epileptogenic electrophysiological properties.^1^ An International League Against Epilepsy (ILAE) consensus classification system for FCD was published in 2011^2^ and an update was proposed in 2022.^3^ FCD type IIIa (FCD IIIa) refers to temporal neocortex alterations in the architectural organization or cytoarchitectural composition, such as cortical dyslamination or hypertrophic neurons outside the layer in the immediate vicinity of hippocampal sclerosis (HS). For instance, HSs exhibit predominantly horizontal architectural abnormalities in the temporal lobe, specifically, the absence of neurons in layers II and III.^2^ Good interobserver and intraobserver agreement in the evaluation of the ILAE classification of FCDs has been achieved among expert neuropathologists.^4^

The standard treatment for intractable seizures related to FCD is the surgical removal of the lesion.^5^ FCD IIIa, a relatively common pathological subtype of temporal lobe epilepsy (TLE), has worse postsurgical outcomes than isolated hippocampal sclerosis (iHS).^6^ Standard anterior temporal lobectomy with amygdalohippocampectomy (ATL) and selective amygdalohippocampectomy (SAH) are effective treatment strategies for intractable TLE. Both the ATL and SAH approaches have comparable positive impacts on seizure control, whereas SAH tends to reduce cognitive deterioration,^7^ auditory naming^8^ and visual field deficits^9^ after surgery. Further effort should be made during presurgical evaluation to precisely identify FCD IIIa so that the most suitable resection manner can be chosen and postoperative outcomes can be predicted. Nevertheless, slight alterations in the temporal neocortex of FCD IIIa patients pose a challenge for the preoperative diagnosis and definition of the resection range.

The landscape of gene expression changes across all cell types in resected FCD brain lesions has been studied.^10^ However, due to the lack of information on specific cell types, the bulk sequencing and averaging of gene expression in tissue samples from all types of neurons, glial cells and nonneural cells has resulted in signal dilution.^11^ A comprehensive molecular atlas of human and mouse brain and cortical development revealed diverse cortical cell types with domain‐specific regulatory mechanisms and genomic interplay in different neurogenic areas of the brain.^12^, ^13^ Importantly, parvalbumin (PV)‐positive GABAergic interneurons were selectively impaired in gene expression in the cortex of patients with FCD.^14^ Several studies have shown that certain neuronal subtypes contribute more to seizure development and contagion than others, and not all subtypes of neurons are equally affected in epilepsy.^15^ Single‐cell sequencing is a powerful technique for unbiasedly evaluating large‐scale simultaneous profiling of numerous molecular characteristics in individual cells and has a great advantage in revealing the cellular diversity and heterogeneity of tissues at single‐cell resolution.^16^ The emerging field of multimodal single‐cell dataset integration and analysis has great potential to enhance our comprehension and understanding of heterogeneous cell populations within diseased brains because it focuses not only on studying gene expression but also on exploring the transcriptional regulatory mechanisms and spatiotemporal transcriptional characteristics.^17^

Given the complexity of FCD IIIa, it is crucial to uncover the function of each cell type in the diseased brain, necessitating single‐cell resolution. Multimodal integration of single‐nucleus RNA sequencing (snRNA‐seq) and single‐nucleus assays for transposase‐accessible chromatin sequencing (snATAC‐seq) allows for a comprehensive understanding of how transcription factor (TF) and chromatin regulate and control the expression of neighbouring genes.^18^ We conducted snRNA‐seq and snATAC‐seq on identical human brain tissue samples to examine cell type‐specific changes in chromatin accessibility and linked them to gene expression changes in the epileptogenic cortex of FCD IIIa. Our integrated multiomic analysis provides a distinct perspective on the range of cellular diversity that contributes to the development of this disease.

## 2 MATERIALS AND METHODS

### 2.1 Human sample collection

The study was approved by the Medical Ethics Committee o of the Seventh Affiliated Hospital of Sun Yat‐sen University, Shenzhen (no. KY‐2023‐019‐02). Informed consent was obtained from all individual participants included in the study. Highly epileptogenic neocortical tissue was obtained from four patients with FCD IIIa. In addition, three TLE patients with relatively nonepileptic and no obvious histological aberration in the temporal neocortex were chosen as controls.^19^, ^20^, ^21^ Our research focused on temporal neocortical regions because the severe degeneration of hippocampal tissue in FCD IIIa patients hinders comparative transcriptomic and epigenetic analysis.^22^ Additionally, data from simultaneous recordings of the hippocampus and temporal neocortex strongly indicate that abnormal tissue in the temporal neocortex is frequently epileptogenic.23 All participants recruited for the study were subjected to standardized preoperative assessment procedures before undergoing standard ATL, including a detailed medical history, neurological and neuropsychological examinations, high‐resolution magnetic resonance imaging (3.0T), video electroencephalographic, and 18‐fluorodeoxyglucose positron emission tomography. The detailed clinical characteristics of the enrolled subjects are provided (Supporting Information Data S1: Data S1). The highly epileptogenic temporal neocortical tissue was localized by preoperative stereoscopic electroencephalograph (SEEG; Figure S1A) and reconfirmed by intraoperative electrocorticogram (ECoG) recording (Figure S1B).^24^ As intraoperative ECoG is crucial for identifying this intrinsically epileptogenic dysplastic cortical tissue,^25^ all patients underwent standardized intraoperative ECoG^26^ under standardized general anaesthesia^27^ immediately prior to ATL. Highly epileptogenic activity was considered to be present when a consistent morphology^25^ and distribution at >1 spike per 10 s was observed in the ECoG recordings.^25^, ^28^ The controls were characterized by no epileptic discharge based on SEEG and ECoG recording and no obvious histological aberration. All patients were verified by postsurgical histological analysis (Figure S1C). Neuropathological assessments included assessment by hematoxylin and eosin (HE), NeuN, GFAP, SIM‐32, CD34, NF, and Vimentin.^29^ Neuropathological evaluation was conducted by two neuropathologists who reviewed more than 30 epilepsy surgery tissues monthly. Patients with highly consistent diagnostic opinions from two neuropathologists were selected for inclusion. FCD IIIa was determined using the ILAE criteria.^2^, ^3^ To ensure that the samples were fresh enough, brain tissues were collected within 10 min after resection and stored in liquid nitrogen until use.

### Brain tissue dissection and cell dissociation

The process of nucleus isolation was conducted according to the 10xGenomics protocol but with some revisions. All steps were carried out on ice. RNA integrity was assessed using a 2100 Bioanalyzer (Agilent). Each brain tissue sample was cut into small pieces and then transferred to a Dounce homogenizer with 400 µL of lysis buffer (pH 7.4, 10 mM Tris‐HCL, 3 mM MgCl2, 10 mM NaCl,.1% IGEPAL,.1% Tween 20, 2% BSA). To release the nuclei, the tissues were homogenized. After 10 min of centrifugation at 4°C, the nuclei were washed in 1 mL of cold nuclei wash and resuspension buffer (1× PBS supplemented with.2 U/µL RNase inhibitor and 1% BSA). Trypan blue and Countess II FL Automated Cell Counter/Microscopy were used to evaluate lysis efficiency and viability. A 40 µm filter was used for the elimination of large clumps and cell debris. Another centrifugation step was performed, and the nuclei were collected for direct myelin removal. Then, 500 µL or an appropriate volume of nucleus wash and resuspension buffer was added to the nucleus pellet to reach the desired 700–1200 nuclei/µL concentration. The 10xGenomics protocol was then used to produce snRNA‐seq and snATAC‐seq libraries.

### snRNA‐seq

We loaded the nucleus suspension into a Chromium microfluidic chip with 3′ (v2 or v3) chemical properties and labelled it with barcodes using a 10xChromium controller (10xGenomics). Following reverse transcription of RNA from the cells labelled with barcodes, sequencing libraries were prepared using reagents from a Chromium Single Cell 3′ v2 or v3 reagent kit (10xGenomics) following the manufacturer's guidelines. Sequencing was carried out with Illumina NovaSeq xplus following the guidelines provided by the manufacturer (Illumina).

### snATAC‐seq

We loaded the nucleus suspension into the Chromium Next GEM Chip H with 10x reagents and labelled it with barcodes using a 10×Chromium Controller (10X Genomics). Following the amplification of DNA fragments from the cells labelled with barcodes, sequencing libraries were prepared using reagents from the Chromium NextGEM Single Cell ATAC Reagent Kit v1.1 (10x Genomics) following the manufacturer's guidelines. The scATAC‐seq libraries were constructed following the Chromium NextGEM Single Cell ATAC Reagent Kit v1.1 User Guide RevC (10x Genomics CG000209). Then, we pooled the libraries and loaded them onto an Illumina NovaSeq 6000 instrument with 2 × 50 paired‐end kits at Novogene.

### Quality control for snRNA‐seq

FASTQ was utilized for conducting fundamental quality statistical analysis on the raw reads. Typically, Cell Ranger counts can process FASTQ files derived from raw base call files produced by Illumina sequencers as input files. We decomposed and mapped the raw reads to the reference genome through the 10xGenomics Cell Ranger pipeline with default parameters for cell and UMI counting and generation of a cell gene expression matrix. Cell Ranger and Seurat were utilized for all subsequent single‐cell analyses unless otherwise specified. Seurat performed additional filtering where a gene was deemed expressed if it was found in more than three cells, and each cell had to express at least 200 genes. We removed nonnative cells based on indicators such as mitochondrial and haemoglobin UMI proportions. We also removed multiple cells from the dataset. On average, we sequenced to a depth of almost 350 million reads per sample for snRNA‐seq. The median gene count across nuclei ranged from 2201 to 2339 genes in each sample. UMI counts were approximately twice the gene count for all samples, as expected for this level of sequencing depth. Between sample groups, there were no significant differences between cases and controls in the number of cells detected per individual, the median gene count per nucleus, the total genes detected per individual, or the median UMI count per nucleus. More than 88% of the nuclei passing these filtering criteria for mitochondrially encoded genes. These underscore the high quality of our snRNA‐seq dataset. After quality control, we obtained 88 158 high‐quality nuclei for snRNA‐seq, including 48 744 from patients with FCD IIIa and 39,414 from controls (Figure S2A.B; Table S1). The uniform manifold approximation and projection (UMAP) approach was applied to preprocess and normalize the snRNA‐seq data for unsupervised analysis. Clusters did not appear to be influenced primarily by batch, sex, age, course, or subject (Figure S3).

### Quality control for snATAC‐seq

The raw sequencing data were converted to FASTQ format with the use of cellranger‐atac mkfastq. snRNA‐seq reads were mapped to the reference genome and quantified with a cellranger‐atac count (10x Genomics) using default parameters, after which peak identification and cell filtration were performed to generate a peak cell count matrix. The quality of the snATAC‐seq dataset of the human temporal cortex was confirmed by expected fragment size distributions, high enrichment of unique open chromatin fragments at transcription start sites (TSSs), good separation between the cell and non‐cell associated barcodes, ideal distribution of fragment quantity in barcode and high fraction of fragments overlapping targeted region. We identified and removed inferior‐quality nucleus from the snATAC‐seq dataset according to the following criteria: fragments mapped to peak regions, reads mapped to peaks, reads mapped to encyclopedia of DNA elements (ENCODE) blacklisted regions, a nucleosome signal, and the enrichment of TSS. Using ArchR software was used for dual‐cell removal. A total of 63 109 nuclei for snATAC‐seq were retained for cross‐sample integration analysis. (Figure S2C–F; Table S2).

### Cell type‐specific dimensionality reduction and cluster analysis

For snRNA‐seq, Seurat applies principal component analysis (PCA) to reduce the dimensionality of multidimensional data and transfers the reduced PCA data to t‐distributed stochastic neighbour embedding and UMAP for two‐dimensional visualization. Seurat uses graph‐based and K‐means methods for clustering cells with similar expressions. For snATAC‐seq, Signac standardizes the data using the term frequency‐inverse document frequency method, which is performed separately for the cell and peak dimensions. Then, feature selection and singular value decomposition (similar to transcriptome PCA) were conducted to generate a low‐dimensional matrix. Finally, graph‐based clustering and UMAP visualization were performed based on this matrix.

### Annotation of cell subpopulations

Canonical marker gene signals were manually inspected to assign major cell type annotations to UMAP partitions and initial clusters in the snRNA‐seq and snATAC‐seq datasets. Similar to the major cell types, snRNA‐seq subpopulations were annotated by utilizing canonical marker gene signals and differentially expressed genes. The snRNA‐seq subpopulations were annotated through hierarchical clustering using the gene expression matrix of the top 25 differentially expressed genes (DEGs) from each subpopulation, ranked by average log fold change (logFC). Seurat label transfer prediction scores were utilized to annotate the snATAC‐seq subpopulations, referencing the snRNA‐seq clusters for annotation.

### Integrated analysis of the snRNA‐seq and snATAC‐seq data

The cell types in the snATAC‐seq data were predicted by using cells from the compatible snRNA‐seq analysis as a reference dataset. The variable features of the snRNA‐seq data were used as a reference for this prediction, while Seurat's GeneActivity was used to generate the gene activity matrix, which served as the query data, from the snATAC‐Seq data. FindTransferAnchors was used to learn transfer anchors, and TransferData was used to predict cell type labels with the snATAC‐seq LSI reduction data as the input for weight reduction. Each cell in the snATAC‐seq dataset was assigned a cell type or subcluster identity using information from the corresponding snRNA‐seq data determined by the first 30 LSI components amended by Harmony, with the exception of the first one. Cells with a prediction score greater than.5 were retained for the following analysis. The prediction score was recorded as the prediction.score.max, which quantifies the level of uncertainty in the predicted annotations. We further subdivided the cell clusters transferred from the snRNA‐seq data into subclusters if the independent snATAC‐seq data supported unsupervised clustering analysis.

### High dimensional weighted gene coexpression network analysis

The R WGCNA package was utilized to conduct high dimensional weighted gene coexpression network analysis (hdWGCNA). The process of constructing coexpression networks closely resembled that of a prior study. The WGCNA package utilized 12853 genes, applying a power of 8, a minModuleSize of 50, a reassignThreshold of 0, a mergeCutHeight of.2, and a scale‐free model fit cutoff of.9. Initially, genes with low variance (variance > 3) were excluded to eliminate features with low expression, resulting in 3394 remaining genes. Then, we normalized and clustered the read counts of the 3394 genes to inspect potential obvious outliers. Then, based on a soft thresholding power of 4, we constructed a signed weighted correlation network and identified the modules. A mergeCutHeight parameter of.2 was utilized for module merging. We calculated the adjacency of eigengenes based on their correlation to further explore the coexpression similarity among all the modules. Module eigengenes, which are also referred to as module eigengene‐based connectivity kME, were utilized for assessing the module membership. Intramodular hub genes (kME > .8) were defined as genes with maximum module membership values. The hub genes within the module are concentrated within the module, representing the expression patterns of the entire module. The top 10% of the most connected genes in each module were identified as hub genes. The hub genes were used to create network diagrams in Cytoscape (version 3.6.1) for visualization of different modules. Geneontology biological processes (GO‐BP) enrichment analysis was performed on the module hub genes using the same method used for the DEGs.

### Identification of TFs and TFs‐hub gene regulatory network construction

pySCENIC (version 0.10.0), a tool for single‐cell regulatory network inference and clustering, was used to calculate the possible GRNs regulating excitatory neurons (ENs) and oligodendrocyte precursor cells (OPCs). Using GENIE3, we deduced the regulons comprising TFs located upstream and their potential downstream target genes. Then, RcisTarget was used to analyse these regulons. Target genes representing notable motif enrichment of matched TFs were preserved. The TF motif search was restricted to genomic regions within a 10 kb radius of the TSS. Subsequently, we utilized AUCell to assess the activity of each regulon in each nucleus, to determine the active regulons in different types of nucleus. Next, we intersected TFs and regulons that were determined to be differently enriched in pySCENIC and activity motifs identified differently via snATAC‐seq and selected overlapping TFs that were reconfirmed via the JASPAR2020 database.^30^ A TF‐hub gene regulatory network was constructed of the ENs.

### Pseudotime trajectory analysis of OPCs

Using the Monocle 2 R package (v2.12.0), single‐cell pseudotime trajectories of OPCs were constructed to identify developmental conversions. Briefly, Monocle was used to decrease the space to a single space and organize the cells; then, visualization was provided via the trajectory in the reduced‐dimensional space. Seurat identified highly variable genes to arrange cells in order of pseudotime. All cell pseudotime values were calculated based on their biological importance, and the most original cell cluster in the differentiation state was identified as the starting point of pseudotime in the first round of “orderCells”. Then, “orderCells” was called again, and this status was transmitted as the root status argument. We applied “DDRTree” and “UMAP” to decrease the dimensional space, followed by plotting the minimum spanning tree on cells with the visualization functions “plot complex cell trajectory”.

### Construction of the ligand–receptor interaction map

To evaluate potential intercellular communication networks in snRNA‐seq data across ENs and OPCs subclusters, we used the CellChat (version 1.6.1) platform implemented in R software with default parameters. Initially, the gene expression data were projected onto a protein–protein interaction network, and then probability values were assigned to predict biological intercellular communication networks using the computeCommunProbPathway and aggregateNet functions. Subsequently, we calculated the centrality indices of the interaction network to clarify the role and division of each cell population in diverse signalling pathways. Additionally, the netVisual_circle function was used to analyse and visualize the intercellular communication network and network strength. Finally, utilizing the human ligand–receptor pairs database, the intercellular communications and visualization of ligand–receptor interplay intensity among different cell populations were inspected by the netVisual_bubble function.

### Western blotting

The brain samples were separated on 4–12% sodium dodecyl sulfate—polyacrylamide gels, electroblotted onto PVDF membrane (Merck Millipore) and stained with primary antibodies against ZEB1 (Abcam) and DAB1 (Santa Cruz), followed by incubation with anti‐mouse or anti‐rabbit horseradish peroxidase‐linked antibody (Cell Signaling). Signals were visualized with an enhanced ECL system (Merck Millipore). Relative protein expression was then normalized by a specific antibody against β actin (Cell Signaling).

### Immunofluorescence

Five‐micron‐thick paraformaldehyde‐fixed paraffin‐embedded sections were cut. After dewaxing and blocking, DAB1 and ZEB1 primary antibodies (antibody concentration 1:500) were added and incubated overnight. Fluorescently labelled secondary antibodies were used. After sealing with the DAPI solution, the slices were scanned using a fluorescence scanner, and the images were semiquantitatively analysed by Image J software.

### Statistical analysis

Statistical analysis was conducted with the Statistical Package for the Social Sciences (SPSS) 25.0 software (IBM). Continuous variables are presented as either the mean ± standard deviation or the median, and categorical variables are shown as counts. Continuous variables between the two groups were compared and analysed with the independent *t*‐test or Wilcoxon rank‐sum test. A two‐sided *p*‐value < .05 was considered to indicate statistical significance, and *p*‐values without significant differences are not displayed.

## 3 RESULTS

### 3.1 Multiomics study of cellular heterogeneity in the human temporal neocortex

**FIGURE 1:**
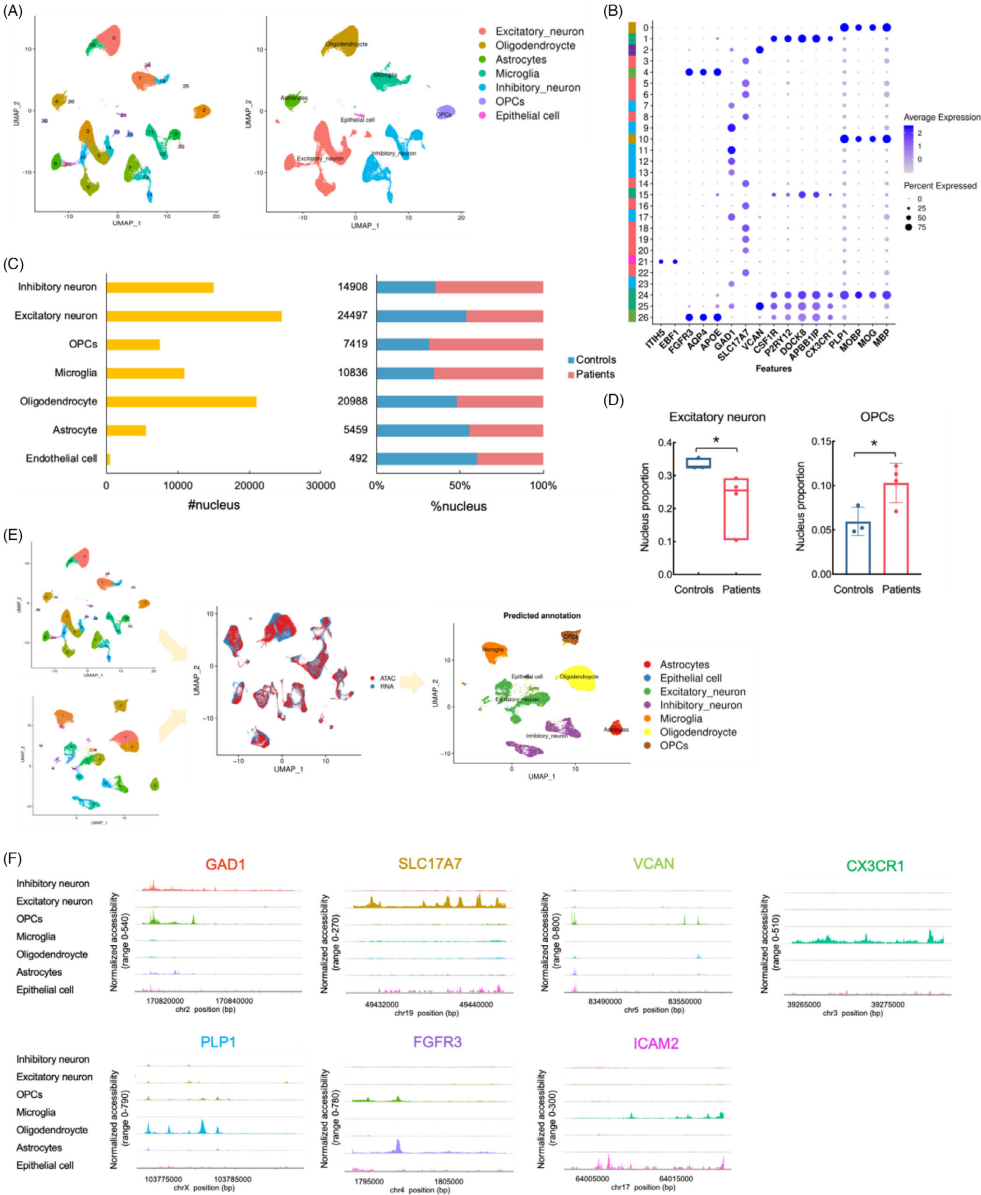
Multiomics study of cellular heterogeneity in the human temporal neocortex. (A) UMAP representation of the snRNA‐seq dataset, which is coloured based on cluster assignment and cell type. (B) Dot plot showing the gene expression patterns of subtype‐specific markers for principal cells in the snRNA‐seq dataset. Rows and columns represent marker genes and subtypes, respectively. The intensity of the dot colour represents the average expression of this marker in a given subtype relative to the other subtypes. The size of the dot reflects the percentage of cells that express the indicated gene. (C) The number of identified nuclei in each cell type, as well as the ratio of profiled cells from controls and patients in each cell type. Compositional changes across conditions are shown. (D) For the nucleus proportion, a notable decrease in ENs and increase in OPCs were observed for patients. The independent *t*‐test and the Wilcoxon rank‐sum test were used for the comparison of continuous variables between the two groups. A two‐sided *p* < .05 was considered significant. ENs, *z* = −2.121. OPCs, *t* = −2.865, *p < .05. (E) Multiomics integration strategy for processing of the snATAC‐seq data. After integrating and transferring labels, the snATAC‐ seq data were assigned according to the predicted cell type. The snATAC‐seq dataset is coloured according to cell type assignment. (F) Pseudobulk profiles of chromatin accessibility for each classic cell type marker gene.

The snRNA‐seq and snATAC‐seq were conducted (10x Genomics) on nuclei isolated from the temporal cortex, which included all cortical layers, using fresh human tissue from the FCD IIIa patients and controls. Transcriptomic and epigenetic datasets were specifically generated from the equally divided samples from the same brain tissue to further minimize variations in cell type composition between the two sequencing approaches, thus allowing for meaningful downstream integrated analysis. After first‐round clustering, unsupervised clustering of the cells from the snRNA‐seq data identified 28 clusters across the seven temporal cortex samples from the FCD IIIa patients and controls (Figure S4A). Of the 28 clusters, that demonstrated high heterogeneity in the temporal cortex, 27 could each be confidently annotated according to the expression of classic gene markers for one of the seven major brain cell types (Figure 1A): ENs (27.79% of nuclei), inhibitory neurons (16.91% of nuclei), oligodendrocytes (23.81% of nuclei), astrocytes (6.19% of nuclei), OPCs (8.42% of nuclei), endothelial cells (.56% of nuclei), and microglia (12.29% of nuclei). An unassigned cluster did not display the expression profile of neuronal or glial marker genes (4.04% of nuclei) (Figure S4B). Multiple forms of cell‐type cluster‐specific gene expression patterns were visualized, including a dot plot (Figure 1B), violin plots and a stacked violin diagram (Figure S5), to form a consensus for annotation. Analysis of the composition of each major cell type (Figure 1C) found that the ratio of nuclei in each subject contributing to ENs (*SLC17A7* ^high^) was significantly lower in the FCD IIIa patients than in the controls, whereas the proportion of OPCs (*VCAN* ^high^) was notably greater in the patients than in the controls (Figure 1D, Table S3). The results indicated that ENs and OPCs are vulnerable to FCD IIIa and have attracted more attention.

Integration analysis of the snATAC‐seq and snRNA‐seq data led to the identification of major cell types with no specific bias. With snATAC‐seq, we identified 21 distinct clusters (Figure S6), and annotated all seven major cell types in the brain (Figure 1E), confirmed by chromatin accessibility upstream of classic marker genes (Figure 1F). The distribution of the number and proportion of nuclei in both datasets is comparable (Table S4). These data not only demonstrated cell characterization in our snATAC‐seq dataset but also showed strong agreement between the snRNA‐seq and snATAC‐seq data.

### 3.2 EN cell‐type‐specific regulation in FCD IIIa

#### 3.2.1 Cell type‐specific transcriptional regulatory networks in ENs

**FIGURE 2:**
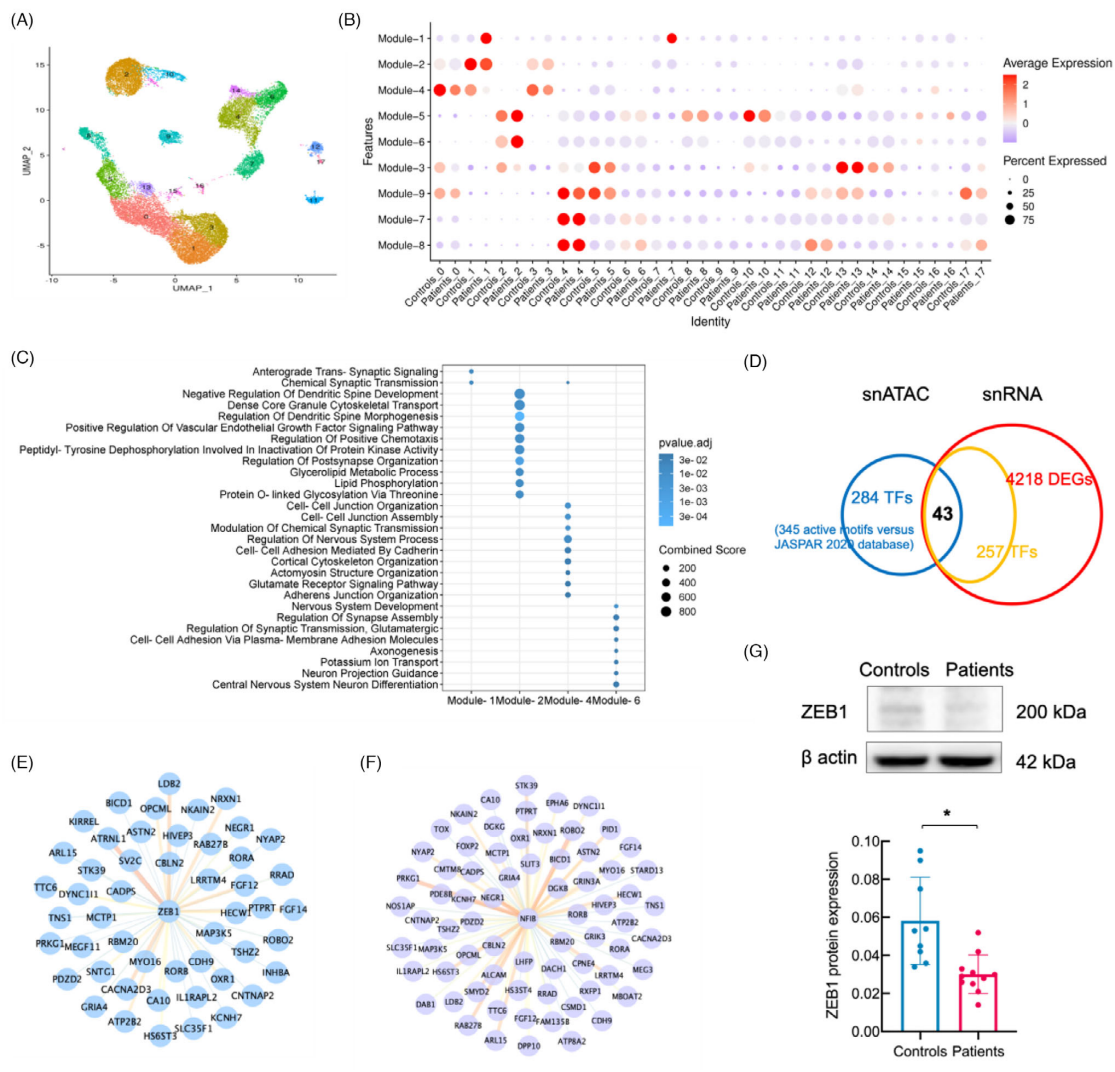
Transcriptomic and epigenetic inference of regulatory biology in EN subtypes. (A) Subclustering of ENs on the UMAP plot of the snRNA‐seq datasets. (B) Dot plot of snRNA‐seq data displaying the expression profiles of modules for controls and FCD IIIa in EN subtypes. Rows and columns represent subtypes and modules, respectively. The intensity of the dot colour represents the average expression of this module in a given subtype relative to the other subtypes. The size of the dot reflects the percentage of cells that express the specified module. (C) Gene ontology (GO) enrichment term of the modules identified by hdWGCNA in ENs. (D) Venn diagrams quantifying the process of key transcription factor recognition. (E) Predicted *ZEB1* regulon networks for the hub genes according to hdWGCNA. (F) Predicted *NFIB* regulon networks for the hub genes according to hdWGCNA. (G) Samples of the brain from the controls and FCD IIIa patients were collected for western blotting analysis. Expression of ZEB1 in the brain of different groups and statistical analysis of the expression ZEB1. Controls *n* = 3, patients *n* = 4. Quantification was conducted on three independent repetitive experiments. The independent *t*‐test. ZEB1, *t* = 3.418. *p < .05.

After second‐round clustering, unsupervised clustering of the ENs from the snRNA‐seq data identified 17 clusters (designated ‘Ex’) (Figure 2A). The hdWGCNA was performed to investigate the coexpression modules and hub genes in ENs. Different nine gene modules (Supporting Information Data S2: Data 2A) and several hub genes (Supporting Information Data S3: Data 2B) were identified. Compared with that in the control group, the most significant change in the FCD IIIa group was the upregulation of Module‐1 in Ex‐1 and Ex‐7. Notably, for Ex‐1 of FCD IIIa, which exhibited the most significant changes in the module, Module‐1 was upregulated and Module‐2 and Module‐4 were downregulated (Figure 2B). The three coexpression modules were the modules with the highest number of genes significantly enriched in GO terms and correlated with FCD IIIa (Figure 2C; Supporting Information Data S4: Data S2C). These findings suggest that Ex‐1 play a crucial role in FCD IIIa.

A total of 305 cell type‐specific TF motifs were identified and confirmed by the JASPAR database.^30^ These motifs exhibited significant differences in activity within the FCD IIIa EN cluster. The DEGs in the snRNA‐seq (Supporting Information Data S5: Data S3A) intersected with the TF with altered motif activity according to snATAC‐seq data (Supporting Information Data S6: Data S3B; Supporting Information Data S7: Data S3C) to identify TFs for which both gene expression and motif activity were significantly different between the patient and control groups. Using this strategy, we identified 43 candidate TFs that likely also play important roles in determining FCD IIIa (Figure 2D). TFs cooperate regularly within transcriptional regulatory networks to ensure the transcriptional output of specific genes. To better understand the regulatory effect of TFs on specific genes in FCD IIIa, we structured cell type‐specific TF regulatory hub gene networks in ENs (Figure S7; Supporting Information Data S8: Data S3D). Among these candidates are many TFs associated with neuronal polarity and migration, such as ZEB1, which regulates the orientation of the cleavage plane of dividing neural progenitors.^31^ Within these networks, we identified multiple hub genes regulated by *ZEB1* (Figure 2E) and *NFIB* (Figure 2F). The motif activity and gene expression of *ZEB1*, *RORA*, and *NR3C2* were decreased in FCD IIIa ENs. We found that *NFIB* and *CUX1* motif activity was increased in FCD IIIa, indicating that more sites are combinable in disease and that the gene expression is also upregulated in FCD IIIa ENs (Supporting Information Data S5, S7: Data S3A, C) We confirmed the decreased (*t*‐test, *p* = .006) protein expression of ZEB1 in the FCD IIIa epileptogenic temporal neocortex by western blotting (Figure 2G).

#### 3.2.2 *DAB1* ^high^ Ex‐1 mediated neuronal immunity characteristically in FCD IIIa

**FIGURE 3:**
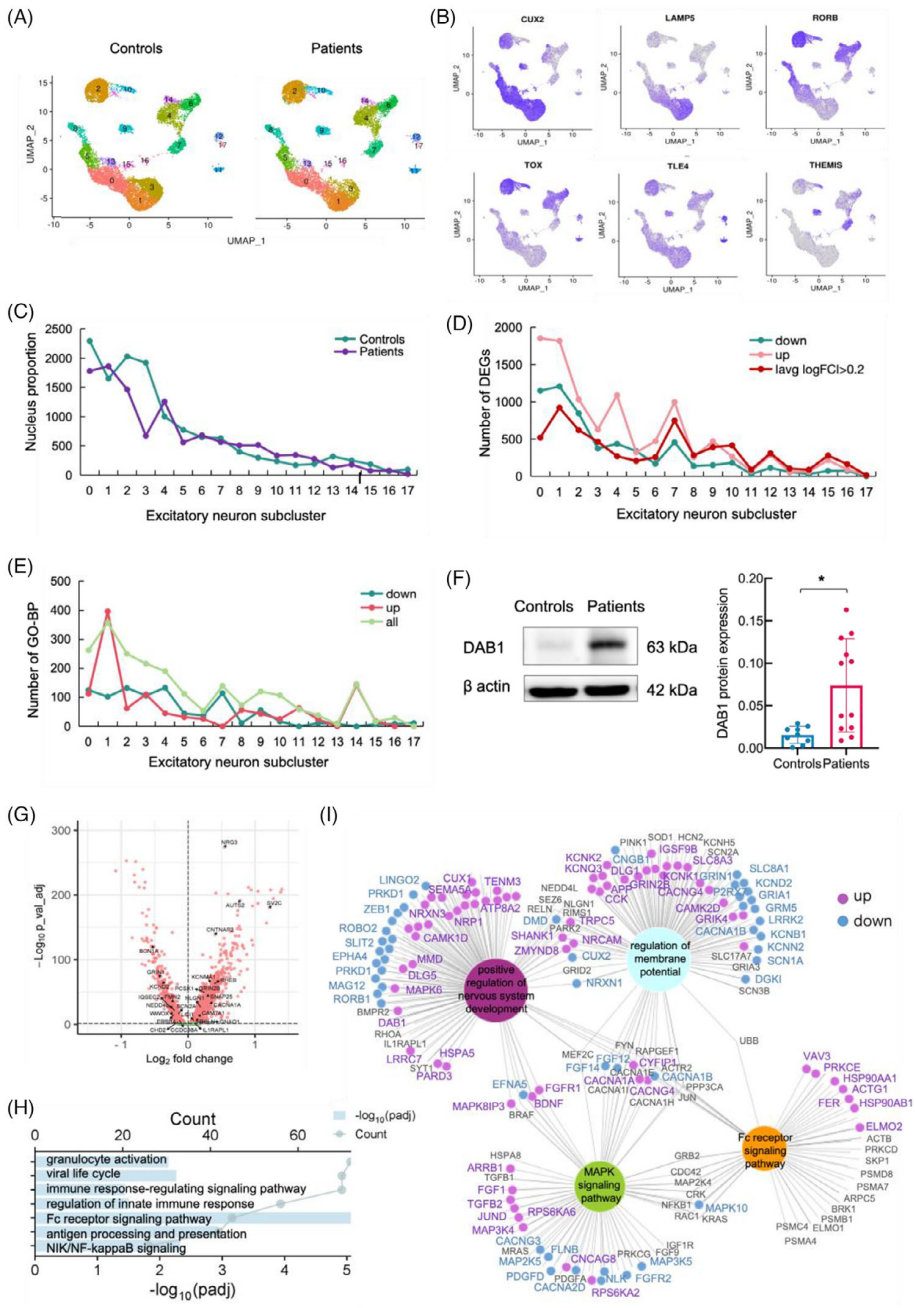
FCD IIIa epileptogenic temporal neocortex is characterized by an increase in *DAB1* ^high^ Ex‐1. (A) UMAP plots of the EN snRNA‐seq dataset for control and patient subjects independently. (B) Expression of the layer marker genes in ENs with a colour scale indicating expression levels. (C) Percentage of nuclei per ENs subtype, showing compositional change across conditions. (D) Number of DEGs per ENs subtype. (E) Number of GO‐BP terms per ENs subtype. (F) Samples of the brain from the controls and FCD IIIa patients were collected for western blotting analysis. Expression of DAB1 in the brain of different group and statistical analysis of the expression DAB1. Controls *n* = 3, patients *n* = 4. Quantification was conducted on three independent repetitive experiments. The independent *t*‐test. DAB1, *t* = −3.601. *p < .05. (G) Volcano plot of DEGs between the FCD IIIa patients and controls in the *DAB1* ^high^ Ex‐1. The enrichment analysis of the DEGs for epilepsy‐associated genes created based on genetics studies of mouse models and human patients confirmed the enrichment of epilepsy genes in *DAB1* ^high^ Ex‐1. (H) The GO‐BP enrichment analysis of the upregulated DEGs in *DAB1* ^high^ Ex‐1, showed several immunity terms enriched. (I) Venn diagram was used to search for shared DEGs from these GO‐BP terms “positive regulation of nervous system development”, “regulation of membrane potential”, “Fc receptor signalling pathway” and KEEG term “MAPK signalling pathway”. Wilcoxon rank‐sum test, adjusted *p*‐value < .05. up, logFC > .2; down, logFC ← .2.

In general, the integration of FCD IIIa and control cortex snRNA‐seq data showed high consistency between the subtypes identified in the two datasets. However, FCD IIIa exhibited a visible increase in nucleus density in Ex‐1 (Figure 3A). Our clustering of ENs coincided with gene expression profiles previously associated with specific cortical layers (Figure 3B). The EN clusters showed a gradient of gene expression. For example, *CUX2*, which is a superficial layer marker for layers II–IV, was mainly detected in clusters Ex‐0, Ex‐1, Ex‐3, Ex‐5, and Ex‐13. Among them, Ex‐1 and Ex‐3 highly expressed the layer II marker gene *LAMP5*. *RORB* is specific to layers III‐V, *TOX*, *TLE* and *THEMIS* for layers V‐VI. However, we observed a decrease in the proportion of nuclei in several *CUX2* ^high^ EN clusters. The Ex‐3 cluster showed a significant decrease in neuronal proportion, indicating the loss of neurons. Interestingly, Ex‐1, located in the same cortex layer as Ex‐3, was the only *CUX2* ^high^ EN cluster with a slightly increased proportion of nuclei (Figure 3C; Table S5). Ex‐1 represents a large population of upper cortical layer neuronal cells and is characterized primarily by the expression of the marker genes *DAB1, CCBE1 and EPHA6* (Supporting Information Data S9: Data 4A), these cells were named *DAB1* ^high^ Ex‐1. These genes are involved in regulating neuronal migration and lamination in the neocortex,^32^ lymphatic sprouting and directional migration,^33^ and regulating neuronal structure during brain development and function.^34^

Our present study suggests that *DAB1* ^high^ Ex‐1 has an aberrant, but essential, role in the epileptogenic cortex of FCD IIIa. There were significant transcriptomic and function changes in *DAB1* ^high^ Ex‐1 between the FCD IIIa group and the control group (Figure 3D) (Supporting Information Data S10: Data 4B). Enrichment analysis of the DEGs in *DAB1* ^high^ Ex‐1 across the GO database highlighted enriched biological process (BP) terms (Figure 3E). Western blotting confirmed the elevated (t‐test, *p* = .004) protein expression of DAB1 in the FCD IIIa epileptogenic temporal neocortex (Figure 3F).

We subsequently analysed DEGs for epilepsy‐associated genes by two gene lists, one created based on genetics studies of mouse models and human patients^22^ (Figure 3G) (Supporting Information Data S11: Data 4C), another originating from BrainBase (Supporting Information Data S12: Data 4D), which confirmed the enrichment of genes from the two epilepsy gene lists in *DAB1* ^high^ Ex‐1. The Kyoto Encyclopedia of Genes and Genomes enrichment analysis of DEGs revealed a wide range of pathway changes, mainly involving the MAPK signalling pathway, Hippo signalling pathway, Rap1 signalling pathway, and cAMP signalling pathway (Supporting Information Data S14: Data 4F). The MAPK pathway was previously reported to be tightly linked to FCD type III.^35^ The GO‐BP enrichment analysis of up‐regulated DEGs indicated *DAB1* ^high^ Ex‐1 enriched several immunity terms (Figure 3H). Interestingly, these terms were absent in other ENs subpopulations. To elucidate how *DAB1* ^high^ Ex‐1 mediated neuronal immunity and contributed to the pathophysiology of FCD IIIa, we explored the relevant GO‐BP terms involved in cortical developmental disorders and epilepsy. The GO terms “positive regulation of nervous system development”, that enrichment of the genes associated with *DAB1* and *ZEB1*, and “regulation of membrane potential” as well as “Fc receptor signalling pathway”, and KEEG term “MAPK signalling pathway” were chosen to search for shared DEGs from these terms (Figure 3I). There was a complex dysregulation of expression for these related genes. The *DAB1* ^high^ Ex‐1 specificity mediated neuronal immunity, which may be involved in the pathogenesis of FCD IIIa.

#### 3.2.3 Validation of gene expression changes

**FIGURE 4:**
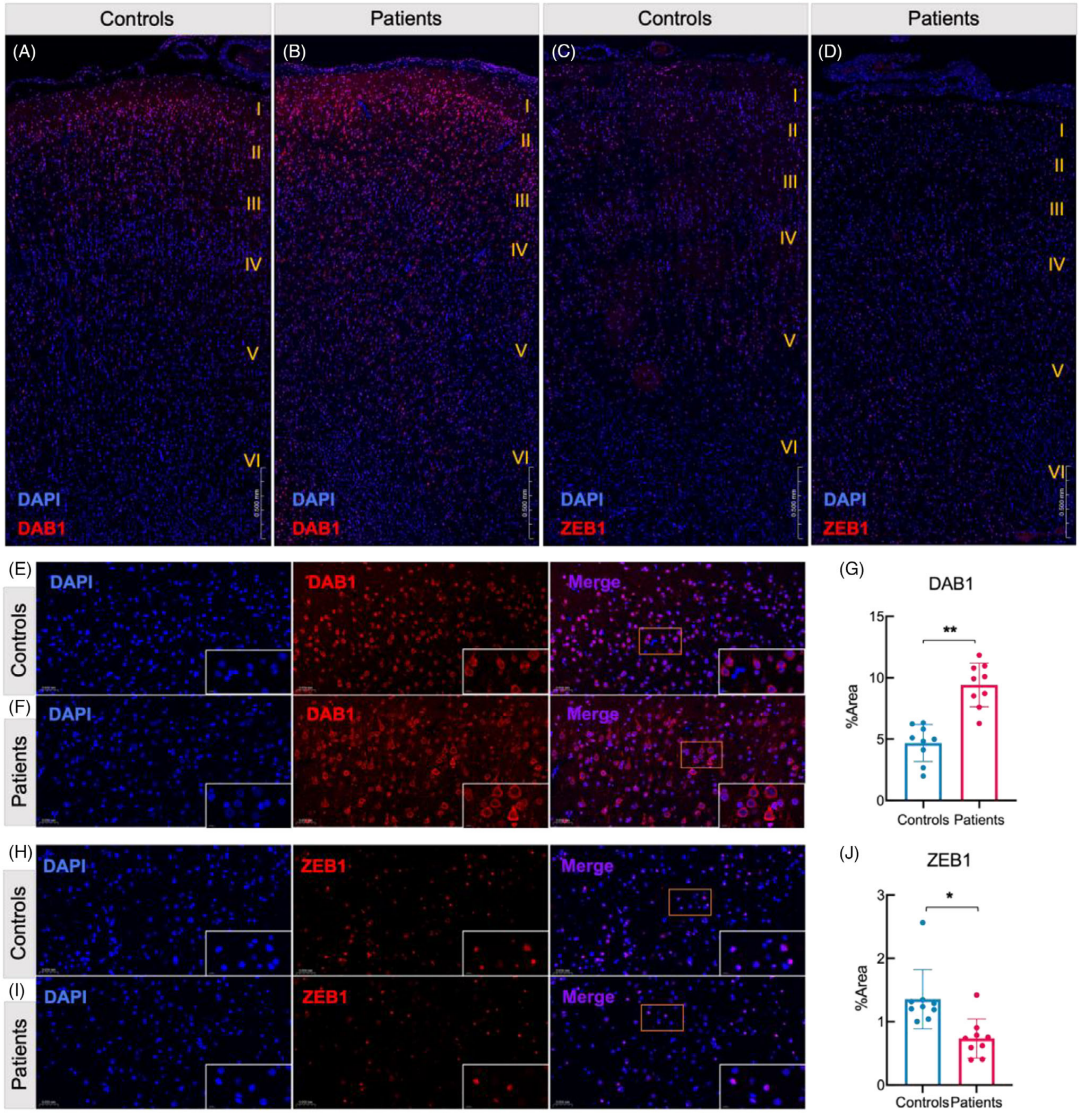
Dysregulation of DAB1 and ZEB1 protein expression in the cortex of patients with FCD IIIa. (A, B) Overview of brain sections from controls (A) and patients (B) showing increased expression of DAB1 in the FCD IIIa, especially in layers II. (C, D) Overview of brain sections from controls (C) and patients (D) showing a decreased expression level of ZEB1 in the FCD IIIa. (E, F, H, I) Representative immunofluorescence images of DAB1 (E, F) and ZEB1 (H, I) in the temporal cortices of control (E.H) and FCD IIIa (F, I) brain layers II. (G, J) Quantification showing the dysregulation of DAB1 and ZEB1 protein expression in the FCD IIIa temporal cortex in layers II. Quantification was conducted on nine randomly selected images from three sections from controls and patients. Controls *n* = 3, patients *n* = 3. The independent *t*‐test. DAB1, *t* = −6.867. ZEB1, *t* = 2.654. **p < .001, *p < .05.

To prove these findings with an independent approach, we performed immunofluorescence on sections of the temporal neocortex that had been fixed in paraformaldehyde and embedded in paraffin. We confirmed the elevated expression of DAB1 (Figure 4A,B) and a reduction in the ZEB1 positive area (Figure 4C,D) in the FCD IIIa epileptogenic temporal neocortex, as determined by labelling antibodies against these proteins. These DAB1 increases and ZEB1 decreases were more obvious in layer II than in the other temporal neocortical regions (Figure 4E,F,H,I). Automated image analysis revealed an increase in DAB1‐positive areas (*t*‐test, *p* = .000) and a decrease in ZEB1‐positive (*t*‐test, *p* = .036) areas in the FCD IIIa group compared with the control group (Figure 3G,J).

### 3.3 OPCs type‐specific regulation in FCD IIIa

#### 3.3.1 OPCs heterogeneity increased in patients with FCD IIIa

**FIGURE 5:**
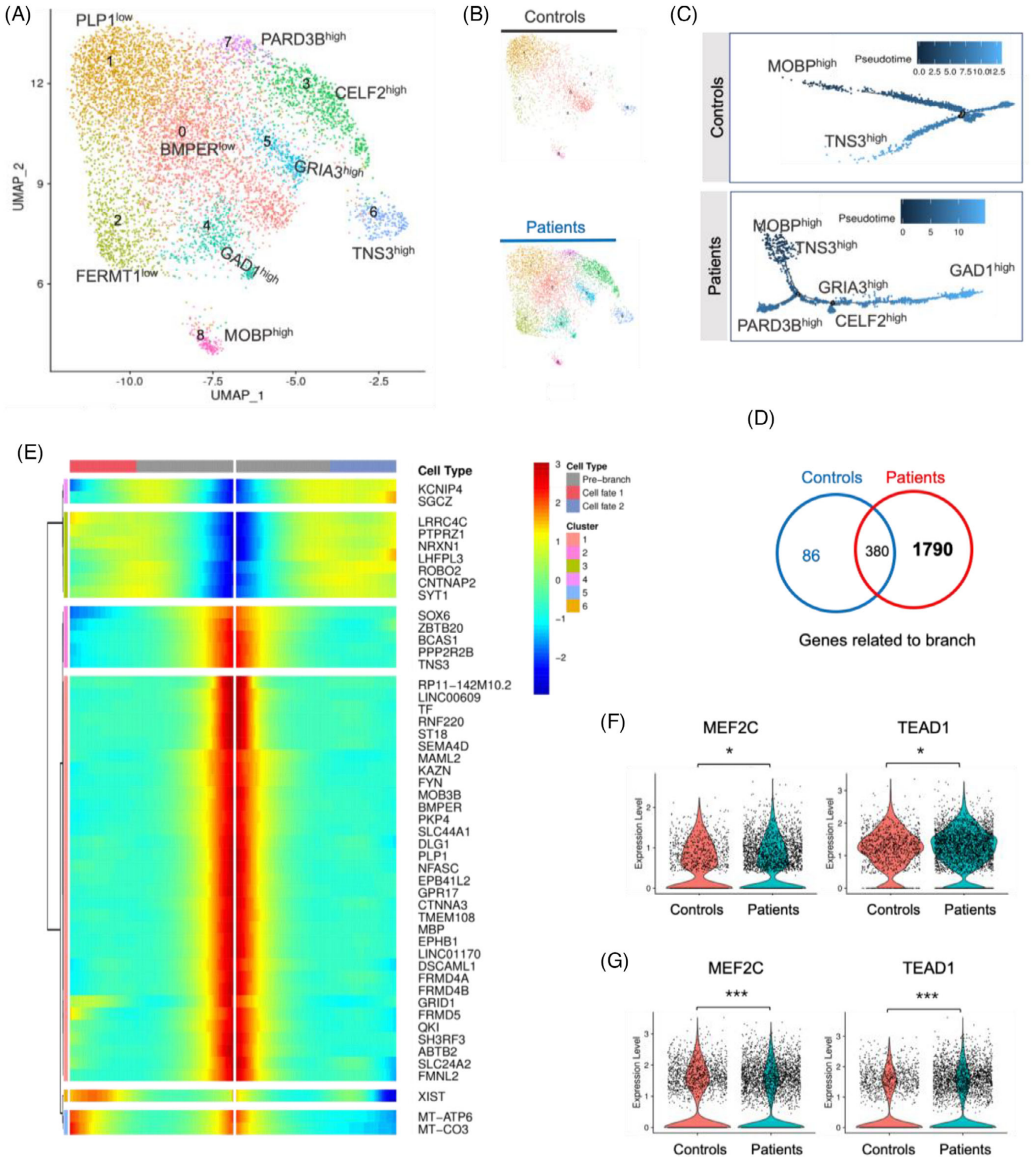
The FCD IIIa epileptogenic neocortex is characterized by increased heterogeneity and reconstructed pseudotime trajectory of OPCs. (A) OPC subpopulations identified with a typical marker gene. (B) UMAP projection and clustering of snRNA‐seq profiles of OPCs from control and patient subjects. (C) The inferred trajectory of OPC subpopulations and their pseudotime ordering was observed in controls, and trajectory reconstruction and pseudotime representation were performed in FCD IIIa. (D) Venn diagrams quantifying the intersection of genes related to the OPCs trajectory branch between controls and patients. (E) Heatmaps of top 50 significant gene expression along the OPCs dysfunction trajectory in FCD IIIa patients. (F) Violin plots of *MEF2C* and *TEAD1* gene expression in OPCs dominated by diagnosis. Wilcoxon rank‐sum test, adjusted *p*‐value.0055 for *MEF2C* and.0489 for *TEAD1*. (G) Violin plots of *MEF2C* and *TEAD1* gene activity in OPCs dominated by diagnosis. Wilcoxon rank‐sum test, adjusted *p*‐value 2.14E‐15 for *MEF2C* and 2.48E‐09 for *TEAD1*. (F, G) Points represent the expression or activity of individual nuclei.

We subclustered OPCs from the snRNA‐seq data and identified nine subpopulations characterized by the expression of several marker genes to identify glial subpopulations (Figure 5A). In the control group, the two largest subpopulations were characterized by low expression of the myelination‐related gene *PLP1* ^36^ and the endothelial cell regulator BMPER^37^ (Figure 5B). Additionally, there are two small subpopulations characterized by high expression of myelination‐related *MOBP*,^36^ and *TNS3*, which is a key mediator of the transient transformation of OPCs to oligodendroglia.^38^ Specifically, we focused on the shift in OPC states in FCD IIIa, noting a rise in the proportion of cells in clusters 3, 4, 5, and 7 (Figure 5B; Table S6). OPCs‐3 was enriched for genes *CELF2* and *GRIA1* (Supporting Information Data S15: Data 5A), which are important RNA binding proteins,^39^ and AMPA receptor subunits.^40^ Cluster 4 expressed the marker genes *TNC* and *GAD1* (Supporting Information Data S15: Data 5A). TNC are associated with the extracellular matrix and stemness.^41^ *GAD1* is involved in GABA neurotransmitter metabolic signalling pathways.^42^ OPCs‐5 was enriched in the genes *MSI2*, *GRIA3* and *ANO3* (Supporting Information Data S15: Data 5A), which are intrinsically required for maintaining stem cell identity,^43^ encode an AMPA receptor subunit,^44^ and reportedly influence neuronal excitability.^45^ Cluster 7 expressed the polarity genes *PARD3B* ^46^ and *FGFR1* (Supporting Information Data S15: Data 5A), which are critical regulators of vascular development.^47^ Cluster 3, 4, 5 and 7 were almost entirely composed of FCD IIIa cells. Moreover, a total of 473 DEGs were identified between the OPCs of the FCD IIIa group and the control group (Supporting Information Data S16: Data 5B). It suggests that OPCs may be activated and exhibited aberrant phenotypes in FCD IIIa patients.^48^

#### 3.3.2 Pseudotime trajectory reconstruction of OPCs in FCD IIIa

Trajectory analysis enables us to study the dynamics of gene expression throughout the entire process of cell state transformation. To better elucidate the molecular mechanisms that promote OPCs transition and diversity in FCD IIIa, monocle2 was used to perform pseudotime trajectory analysis of the OPCs. Various types of cells were accurately positioned along the predicted trajectory based on their stages of development (Figure S8). Indeed, the distribution of cell types along pseudotime trajectories revealed noticeable shifts in the distribution density in the FCD IIIa patients. Unsupervised trajectory analysis yielded distinctly different trajectory pathways for each group. FCD IIIa cells align with the OPCs activation trajectory and move towards two activation branches, one consisting of *GAD1* ^high^ cells and the other consisting of cells with high *PARD3B* expression (Figure 5C).

We conducted differential gene expression analysis along the pseudotime trajectory in the two groups, and there were marked differences across pseudotime in OPCs developmental genes (Figure 5D; Supporting Information Data S17: Data 6A; Supporting Information Data S18: Data 6B). Using the dysfunction trajectory of OPCs, we can offer a comprehensive perspective of putative dynamic changes in gene expression in FCD IIIa (Figure 5E). Here, we were able to define several new latent genes and transcriptional regulators that distinguish dynamic states. Various time points were observed along the trajectory showing changes in gene expression, such as upregulation of *KCNIP4*, followed by *SGCZ*, *LRRC4C*, *PTPRZ1*, *NRXN1*, *LHFPL3*, *RORB2*, *CNTNAP2*, and *SYT1*, indicating the promotion of cortical development and synaptic transmission alongside the trajectory. The downregulation of *SOX6*, followed by *ZBTB20*, *BCAS1*, *PPP2R2B*, *TNS3*, *BMPER*, *PLP1*, *MBP*, and others, indicated the downregulation of myelination and oligodendroglia transient.

Using the strategy in ENs, we identified candidate TFs that may also be involved in the dysfunction trajectory of OPCs specifically, rather than in controls (Supporting Information Data S19: Data 6C; Supporting Information Data S20: Data 6D). The OPCs of the FCD IIIa group showed downregulated gene expression (Figure 5F) and motif activity (Figure 5G) of *MEF2C*, while *TEAD1* increased. In general, our examination revealed chromatin patterns and regulators associated with disordered OPCs states in patients with FCD IIIa.

### 3.4 Characterization of the intercommunication between ENs and OPCs in FCD IIIa

**FIGURE 6:**
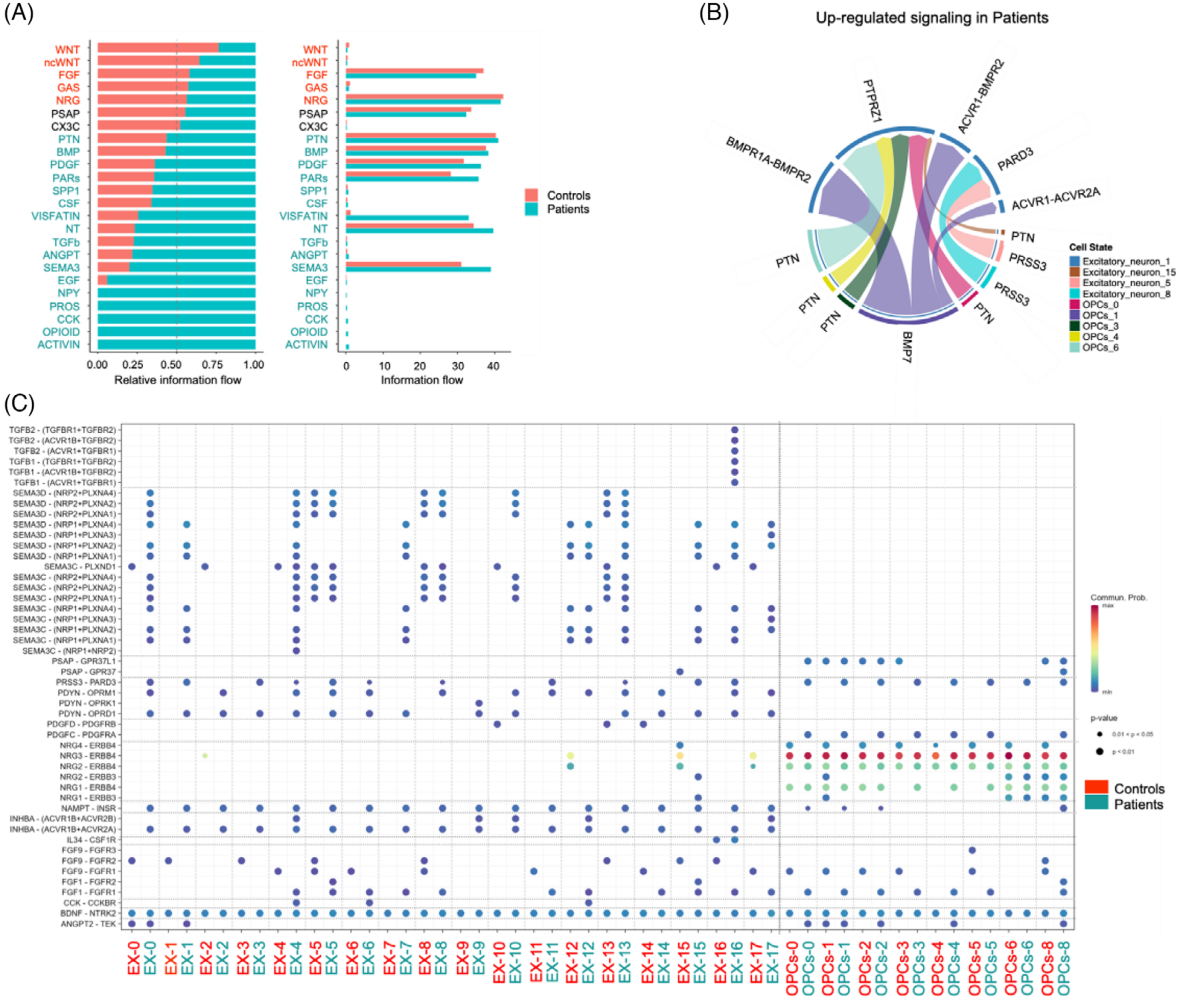
Analysis of intercellular communication between the ENs and OPCs via a receptor–ligand interaction network. (A) Overall altered ligand–receptor interactions between ENs and OPCs in the temporal neocortical region of patients with FCD IIIa detected by Cellchat. On the *y*‐axis, ligand–receptor pairs with no significant difference are shown in black. (B) Chord diagram visualizing the receptor–ligands upregulated by *DAB1* ^high^ Ex‐1 as targets in the disease group. The outer circle represents interacting cell types, with arrows pointing from the source to the target cells. The inner circle represents the intensity of the interaction received by the target cells, and the size of the inner bar is proportional to the intensity of the interaction received by the target cell. The colour of the inner bar is consistent with that of the target cells. (C) Ligand–receptor interactions by *DAB1* ^high^ Ex‐1 as the source, as detected by Cellchat. *x*‐axis, cell types; *y*‐axis, ligand–receptors.

We employed the CellChat program, a tool engineered to visualize intercellular communication networks through the ligand–receptor interactions, to determine the potential intercellular communication dynamics among EN subtypes and OPCs differentially expressed in FCD IIIa lesions versus controls. We compared the overall intercellular signal intensities within disparate signalling pathways between FCD IIIa and control brain tissues. A notable alteration was identified in the interaction of certain ligand–receptor pairs within OPIOID, ACTIVIN, CCK, PROS, NPY, EGF, SEMA3, ANGPT, TGFβ, NT, VISFATIN, CSF, SPP1, PARS, PDGF, BMP, PTN, NRG, GAS, ncWNT, WNT, and FGF (Figure 6A). Additionally, we conducted a thorough analysis of certain ligand–receptor interaction pairs and calculated the differential outgoing and incoming interaction strengths within these signalling pathways (Supporting Information Data S21–23: Data 7A–C). Our analysis revealed that *DAB1* ^high^ Ex‐1 were involved in all of the aforementioned differential signalling pathways, except for NPY, EGF, and SPP1 (Figure 6B,C).

Intercellular communication among ENs becomes dysregulated during FCD IIIa. The OPIOID and ACTIVIN signalling networks were exclusively expressed in the FCD IIIa EN clusters (Figure S9A), possibly because of their abnormal neuronal regeneration^49^ and excitability.^50^ In particular, within the OPIOID and ACTIVIN signalling pathway, *DAB1* ^high^ Ex‐1 and Ex‐16 are the primary sources of PDYN and INHBA, whose receptors were enhanced within all EN clusters (Figure S9B,C). Compared with their control counterparts, the majority of FCD IIIa EN subpopulations showed elevated SEMA3 and VISFATIN signalling (Figure S9A), indicating enhanced priming of adhesion^51^ and neuroprotection.^52^ Moreover, the FCD IIIa *DAB1* ^high^ Ex‐1 cluster exhibited increased CCK, TGFβ, CSF outgoing (Figure 6C) and PROS (Figure 6B) incoming interaction weights and strengths and an impaired ncWNT and WNT cell–cell communication signals.

Intercellular communication between ENs and OPCs is also dysregulated during FCD IIIa. Altered ANGPT, PARS, and PDGF signalling interactions were inferred within FCD IIIa populations, where the EN clusters sent significantly increased signals to OPCs (Figure S9B). ANGPT signalling, which regulates angiogenesis,^52^ was significantly increased in *DAB1* ^high^ Ex‐1 and most OPCs in FCD IIIa. The PARS signal originated from ENs and acted on OPCs, whereas this interaction was intensified in the FCD IIIa group. The function of PRSS3 expressed in the brain is unknown.^53^ The increased PDGF signal, which is one of the central mediators involved in fibrosis,^54^ originates from ENs and OPCs and acts on OPCs. BMP and PTN signals originate from OPCs and act on ENs and OPCs (Figure S9B,C). BMP7 promotes the growth of the mammalian cortex by extending the neurogenic period.^55^ PTN, a neurotrophic growth factor, was shown to act as a self‐regulating factor that enhances the formation of oligodendrocytes and the repair of myelin.^56^ FCD IIIa increased the expression of ACVR1, PTPRZ1, and SDC3 in ENs, mainly in *DAB1* ^high^ Ex‐1 (data were not shown). Our data indicated that the FGF signal originated from ENs and OPCs‐8 and acted on ENs and OPCs, and this signal subsequently decreased during FCD IIIa (Figure S9B,C). Increasing evidence indicates that FGF/FGFR signalling plays important roles in numerous processes in embryonic development and adult homeostasis by controlling cellular lineage commitment, differentiation, proliferation, and apoptosis in different cell types.^57^ In addition, the FCD IIIa *DAB1* ^high^ Ex‐1 cluster gained more NT and exhibited impaired NRG and GAS cell–cell communication signals with other ENs and OPCs.

## 4 DISCUSSION

Our study integrated and analysed paired snRNA‐seq and snATAC‐seq data to identify crucial subpopulations of ENs and OPCs in the epileptogenic cortex of FCD IIIa patients and explore their possible pathogenic role in the disease. Furthermore, independent and combined analysis of the transcriptome and chromatin profiles of ENs and OPCs revealed multiple disrupted hub gene regulators and biological pathways. In ENs, we constructed a TF‐hub gene regulatory network and found that *DAB1* ^high^ Ex‐1 mediates neuronal immunity characteristically in FCD IIIa. Immunofluorescence was used to validate the changes in protein expression levels induced by some of the key genes. The increased heterogeneity of OPCs in patients suggests that they were activated and exhibited aberrant phenotypes and TFs regulating reconstructed pseudotime trajectory were identified. Moreover, our results identified that aberrant intercellular communication between ENs and OPCs in FCD IIIa patients. FCDs are congenital developmental lesions, whereas HS is not congenital but an acquired lesion. The temporal relation of changes in the hippocampus and adjacent temporal neocortex thus is uncertain.^23^ The long‐standing debate over whether HS causes chronic epilepsy or epilepsy leads to HS remains incompletely resolved. Our study revealed significant and intricate alterations in the transcriptomes and epigenomes in ENs and OPCs of FCD IIIa patients, shedding light on their cell type‐specific regulation and potential pathogenic involvement in this disorder. This may suggest an important role of temporal neocortex dysplasia in epileptogenesis and HS.

Our results are consistent with a previous study showing the crucial functions of excitatory neurogenic pools in brain development and the promotion of neuronal hyperexcitability postnatally, as indicated by the distinct spatiotemporal expression patterns of mutated genes in both normal and malformed cortical development brains at the single‐cell level.^35^ Interestingly, the *DAB1* ^high^ Ex‐1, a cluster of ENs with unique characteristics, mediated neuronal immunity and had a substantial effect on the biological function of FCD IIIa. Notably, we found that the marker gene *DAB1* was upregulated in *DAB1* ^high^ Ex‐1. DAB1 is best known as an adapter protein for apolipoprotein E receptor 2 and very low‐density lipoprotein receptor (VLDLR), and it plays a crucial role in the Reelin pathway, which controls neuron migration and lamination during development.^58^ Polymorphic ATTTT repeat mutations within the *DAB1* gene are the cause of autosomal‐dominant spinocerebellar ataxia 37 (SCA37).^59^ The mutation disrupts DAB1 expression, leading to a switch in RNA that results in increased Reelin‐DAB1 and PI3K/AKT signalling in the SCA37 cerebellum.^60^ SCA37 neuropathologically shows a serious loss of Purkinje cells along with a high presence of astrogliosis and empty baskets. Additionally, a small group of Purkinje cells are heterotopically dislocated within the cerebellar cortex.^60^ The neuropathologies of FCD IIIa and SCA37 are similar in terms of neuronal loss and ectopia. These results suggest the potential disruption of DAB1 and the Reelin pathway in ENs in FCD IIIa patients, which may be related to the pathology of this disease.

Recently, increasing evidence has supported the prominent role of the immune system in different subtypes of FCD.^61^ Immune dysfunction can impact the formation and function of synapses, leading to neurodevelopmental diseases.^62^ A study with a rat model of MCD has shown that the outbreak of autoimmune response is associated with spontaneous recurrent seizures in the malformed brain.^63^ Our results align with previous studies, revealing the cell and layer‐specific dysfunction of neuronal immunity in FCD IIIa. We also found that *DAB1* ^high^ Ex‐1 mediated neuronal immunity is located in layer II of FCD IIIa, thus suggesting that immune factors might specifically target cells in the neuron loss layer and the neurons may possess differential regulation in immune pathways. In exploration of candidate entries that contribute to immune dysregulation in *DAB1* ^high^ Ex‐1, we found the GO‐BP term “Fc receptor signalling pathway” which is enriched with DEGs associated with *VAV3* and *HSP90AA1*. Fc gamma receptors (FcγR) with signalling competent expressed on developing neurons in the cortex, and antibodies can modify signalling events and affect normal neural development or function by directly activating FcγR in the brain.^64^ The binding to Fc receptors and the subsequent activation of related intracellular signalling pathways determine the effector activity of antibodies and mediate neurotoxicity and neurodegeneration.^65^ Our findings indicate that the *DAB1* ^high^ Ex‐1 is a crucial driver of immunity in FCD IIIa, and may serve as a novel target for immunomodulatory therapies.

OPCs dysfunction is also suspected to contribute to the pathogenesis of FCD IIIa. OPCs are a type of tissue‐resident glial cells that are present throughout the lifetime of the CNS.^66^ OPCs are primarily responsible for generating myelinating oligodendrocytes. However, they also have multiple functions independent of myelination, such as contributing to neuron development, axon regeneration, angiogenesis, the inflammatory response, and glial scar formation and participating in neural circuit remodelling.^66^ OPCs promote neuronal development and direct interneuron migration through unidirectional contact repulsion,^67^ enabling interneurons to reach their appropriate cortical region. During circuit remodelling in mice, OPCs can engulf synapses and potentially influence synaptic connectivity in the brain.^68^ Limited data have indicated that the proliferation and differentiation of OPCs and the formation of myelin sheaths are hindered in FCD IIa patients, potentially contributing to cortical malformation and epileptogenicity.^69^ Mild malformation of cortical development with oligodendroglial hyperplasia and epilepsy (MOGHE) is a recently recognized additional mild form of epileptogenic disorders of MCD.^70^ MOGHE is histopathologically characterized by patchy zones of hypomyelination, and increased oligodendroglial cell densities and heterotopic neurons in the white matter.^71^ In the epileptic temporal lobe cortex of FCD IIIa patients, OPCs significantly proliferated and accompanied by dysfunction of myelination, which may also suggest the mechanism of the histopathology of MOGHE.

On the other hand, OPCs heterogeneity in the FCD IIIa group revealed cells that express genes associated with the AMPA receptor, maintaining stem cell identity and influencing neuronal excitability and vascular development. *VCAN* and *PTPRZ1* encode two principal proteoglycans (PGs), versican and phosphacan. Extracellular PGs can selectively repel glutamatergic axons and adhere to all parts of the neuronal membrane except dendritic spines, which are the basis for axodendritic synapses being glutamatergic and axosomatic synapses being GABAergic.^72^, ^73^, ^74^ Versican regulates neurite growth and neural crest cell migration.^75^ Phosphacan promotes neuron–glial interactions, neurite outgrowth of primary neurons, neuronal differentiation and development, myelination and axonal repair.^76^ The selective loss of dendritic spines or displacement of axosomatic synapses can alter the epileptogenic potential of such individual neurons.^77^ The findings suggest that OPCs may also play a role in FCD IIIa that is independent of their function as precursor cells for oligodendrocytes.

Since TFs orchestrate gene expression programs, they are considered crucial determinants of cell fate in neurodevelopment processes. Chromatin accessibility is essential to TFs in the process of reading out cis‐regulatory DNA sequences. Although transcriptomic data can independently identify TFs, our results were confirmed by integrating epigenetic data because we linked gene expression with chromatin accessibility. Multiomics analysis constructed a TF‐hub gene regulatory network in ENs and identified the TFs that regulate OPC differentiation in the FCD IIIa group. This finding/observation provides critical insights into a deeper understanding of the specific TFs’ effects during the reprogramming processes in FCD IIIa. As reported, ZEB1 is important for regulating the proliferation and differentiation of neuronal progenitor, and neuronal migration in the mouse neocortex.^31^ During neural development, ZEB1 is a potent intracellular molecule that participates in the epithelial–mesenchymal transition process.^78^ Depletion of ZEB1 seriously disturbs the thickness of the cortical layer.^31^ Furthermore, some studies identified TEAD1 as a crucial TF for Schwann cells in axon regeneration and remyelination after nerve injury.^79^ However, the role of TEAD1 in OPCs remains unclear. In the present study, our data suggests that *TEAD1* is upregulated in FCD IIIa, which may indicate a compensatory response of OPCs after neuronal loss.

Finally, our results revealed aberrant intercellular communication between ENs and OPCs in the cerebral cortex of patients with FCD IIIa, which indicates that the *DAB1* ^high^ Ex‐1 and OPCs may interact directly through the expression of matched ligand receptors. We identified pathogenic and protective factors from the standpoint of intercellular communication. One interesting finding is the emergence of new OPIOID signalling networks among ENs in FCD IIIa. The abnormally elevated PDYN levels coincided with dysregulated glutamate transporters and glutamate receptors and altered neuronal excitability.^50^ Furthermore, we identify interactions emanating from *DAB1* ^high^ Ex‐1 to OPCs, which likely play roles in regulating angiogenesis and fibrosis. We also found that the neighbouring ENs and OPCs promote neuroprotection, neuronal regeneration, remyelination, and cortical growth in FCD IIIa, rather than sending additional apoptotic signals. However, determining whether perturbations in this regulatory complexity contribute to FCD IIIa and whether these perturbations can be leveraged as therapeutic targets should be prioritized in future studies.

Our study is not without limitations. It has been previously described that a potential technical limitation of the droplet‐based snRNA‐seq of human brain samples is the ability to capture nonneuronal cells, such as immunocyte.^80^, ^81^ This study may not have sufficiently captured the anticipated diversity within the overall FCD IIIa population. This limitation may result in possible deficiencies in the comprehensiveness of cell types and the analysis of cell–cell interactions, especially between *DAB1* ^high^ Ex‐1 and immune cells. Despite the limitations of droplet‐based snRNA‐seq in detecting genes with low expression, we successfully detected differential gene expression in numerous genes within specific cell populations. Second, we used western blotting and immunofluorescence to validate the changes in protein expression levels caused by some of the key genes. Although the findings are intriguing, further research, such as translating the information into functional studies in vivo to validate the impact of these TFs on FCD IIIa progression, or validating snATAC‐seq data through epigenetic modulation techniques like chromatin immunoprecipitation followed by sequencing could provide further insights into TFs binding and chromatin accessibility in FCD IIIa.

## 5 CONCLUSION

This work provides a valuable resource for understanding the intricate relationship between cells in diseased FCD IIIa brains, which will help evaluate the pathogenesis of cortical dysplasia and epilepsy and explore potential therapeutic targets. Based on the identified dysregulated pathways, exploring potential therapeutic interventions is a key direction for future research. This will provide new ideas and research directions for the treatment of FCD IIIa and also emphasize the clinical translation potential of our research. Although this study has revealed significant changes in the transcriptomes and epigenomes of ENs and OPCs in FCD IIIa patients and their potential pathogenic mechanisms, these findings need to be further verified in vivo models.

## AUTHOR CONTRIBUTIONS

Liemin Zhou, Yingying Liu, Qiang Guo, and Lisen Sui conceived the study and participated in its design. Yingying Liu acquired and analysed the data; Yinchao Li carried out most of the experiments; Yaqian Zhang carried out part of the experiments; Yubao Fang drafted a significant portion of the manuscript; Lei Lei substantively revised the manuscript; Jiabin Yu and Hongping Tan helped to collect subject information. All authors read and approved the final manuscript.

## CONFLICT OF INTEREST STATEMENT

The authors declare no conflict of interest.

## ETHICS APPROVAL

All procedures performed in studies involving human participants were in accordance with the ethical standards of the institutional and/or national research committee and with the 1964 Helsinki Declaration and its later amendments or comparable ethical standards. The study was approved by the Bioethics Committee of the Seventh Affiliated Hospital of Sun Yat‐sen University, Shenzhen (no. KY‐2023‐019‐02). Informed consent was obtained from all individual participants included in the study.

## Supporting information



Supporting Information

Supporting Information

Supporting Information

Supporting Information

Supporting Information

Supporting Information

Supporting Information

Supporting Information

## Data Availability

The RNA‐seq and ATAC‐seq datasets (FASTQ files) and processed data during the current study were deposited in GEO under accession number GSE266308.
