# Supplementary material for: Excitatory neurons and oligodendrocyte precursor cells are vulnerable to focal cortical dysplasia type IIIa as suggested by single‐nucleus multiomics
Source: Clin Transl Med. 2024 Oct 23;14(10):e70072. doi: 10.1002/ctm2.70072 (PMC11497056; doi:10.1002/ctm2.70072)
Supplement: Supplementary file 1 — Supporting Information [file CTM2-14-e70072-s001.docx]

**Supplementary Fig. 1** Localization of the epileptic zone (EZ) in neocortical tissue of FCD IIIa and relatively non-epileptic neocortical tissue of the control group. (A) Presurgical evaluation and confirmation of EZ and control (a1,2). Represents an area of hypometabolism in the left temporal lobe in a PET scan of the FCD IIIa patient with a negative MRI. (a3) The fusion imaging of MRI and PET of the FCD IIIa patient. (a4,5) Represents ictal discharges restricted to the region in the left temporal gyrus from electrode A-D in the SEEG recordings. (B) Reconfirmation of neocortical samples during surgery. (b1) The visual field and the areas of tissue samples used for electrophysiological recording during surgery. (b2-4) Recordings based on ECoG of the superior, middle and inferior temporal gyrus, showed continuous epileptiform discharges and normal background activity in the temporal region. (C) Post-surgery histological examination with FCD IIIa and control. (c1,2,5,6) HE-stained section of hippocampus sclerosis of control (c1,5) and FCD IIIa (c2,6) groups. (c3,4,7,8) HE-stained section of neocortical of temporal lobe of control (c3,7) and FCD IIIa (c4,8) groups. (c3,7) The control shows relatively normal shaped and arranged neurons in the lateral neocortex from patients with iHS. (c4,8) Architectural abnormalities in the temporal lobe of FCD IIIa patients, indicated by the loss of layer II (arrow). The second panel represents the extension of the area in the first panel. Scale bars represent 500 and 200μm for the first and second panels, respectively.
