# Supplementary material for: Excitatory neurons and oligodendrocyte precursor cells are vulnerable to focal cortical dysplasia type IIIa as suggested by single‐nucleus multiomics
Source: Clin Transl Med. 2024 Oct 23;14(10):e70072. doi: 10.1002/ctm2.70072 (PMC11497056; doi:10.1002/ctm2.70072)
Supplement: Supplementary file 2 — Supporting Information [file CTM2-14-e70072-s004.docx]

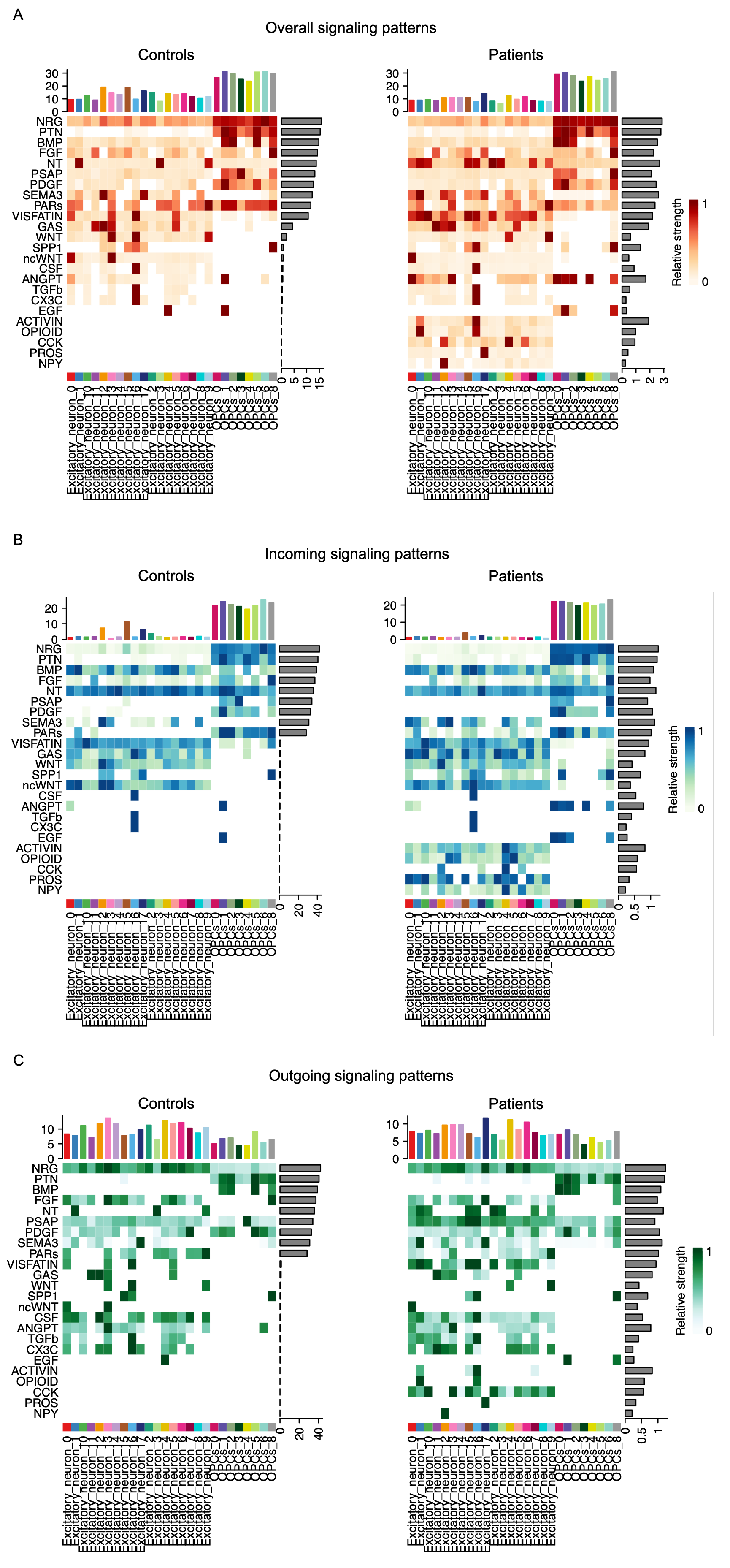


**Supplementary Fig. 9** Heatmap of intercellular communication in ENs and OPCs subpopulations spilt by diagnosis. (A) Overall signaling patterns. (B) Incoming signaling patterns. (C) Out signaling patterns. Heatmap showing intercellular signaling patterns in ENs and OPCs subpopulations. Rows and columns represent subtypes and ligand-receptor pairs, respectively. The intensity of the colour represents relative strength of this ligand-receptor pair in a given intercellular.

| **Supplementary Table 1.** snRNA-seq dataset cell filtering | | | | | | | |
| --- | --- | --- | --- | --- | --- | --- | --- |
| Sample name | Control-1 | Control-2 | Control-3 | Patient-1 | Patient-2 | Patient-3 | Patient-4 |
| Minimum cells | 3 | 3 | 3 | 3 | 3 | 3 | 3 |
| Low thresholds nGene | 200 | 200 | 200 | 200 | 200 | 200 | 200 |
| High thresholds nGene | 6500 | 7000 | 6500 | 6500 | 9000 | 7000 | 6500 |
| High thresholds percent  mitochondrial | 5 | 5 | 5 | 15 | 30 | 15 | 15 |
| High thresholds percent HB | 5 | 5 | 5 | 5 | 5 | 5 | 5 |
| Gene number | 29967 | 29956 | 30004 | 29664 | 29878 | 30193 | 30065 |
| Cell number | 14630 | 14346 | 15339 | 13123 | 11267 | 15089 | 15356 |
| Cell number filtered | 13068 | 12820 | 13526 | 11716 | 10224 | 13286 | 13518 |
| nGene median filtered | 1974 | 2004 | 2055 | 1961 | 2230 | 2036 | 2029 |

| **Supplementary Table 2.** snATAC-seq dataset cell filtering | | | | | | | |
| --- | --- | --- | --- | --- | --- | --- | --- |
| Sample name | Control-1 | Control-2 | Control-3 | Patient-1 | Patient-2 | Patient-3 | Patient-4 |
| Minimun peak region fragments | 200 | 200 | 200 | 200 | 200 | 200 | 200 |
| Maximum peak region fragments | 13500 | 15000 | 11500 | 12500 | 16000 | 14500 | 14500 |
| Minimum perent reads in peaks | 5 | 5 | 5 | 5 | 5 | 5 | 5 |
| Maximum blacklist ratio | 0.05 | 0.05 | 0.05 | 0.05 | 0.05 | 0.05 | 0.05 |
| Maximum nucleosome signal | 4 | 4 | 4 | 4 | 4 | 4 | 4 |
| Minimum TSS enrichment score | 2 | 2 | 2 | 2 | 2 | 2 | 2 |
| Peak number | 168942 | 194608 | 172707 | 183919 | 188505 | 180842 | 178992 |
| Cell number | 8749 | 12813 | 8818 | 7830 | 8517 | 8099 | 8283 |
| Peak number filtered | 168942 | 194605 | 172707 | 183918 | 188505 | 180842 | 178992 |
| Cell number filtered | 8131 | 9585 | 8090 | 7217 | 7897 | 7502 | 7722 |

| **Supplementary Table 3.** snRNA-seq nuclei in each cell-type | | | | | | | |
| --- | --- | --- | --- | --- | --- | --- | --- |
| Sample name | Inhibitory neuron | Excitatory neuron | OPCs | Microglia | Oligodendrocyte | Astrocyte | Endothelial cell |
| Control-1 | 1523 | 4624 | 684 | 1034 | 3856 | 1017 | 91 |
| Control-2 | 1302 | 4164 | 621 | 1006 | 4192 | 857 | 57 |
| Control-3 | 2457 | 4388 | 1051 | 1706 | 2116 | 1171 | 149 |
| Patient-1 | 2541 | 3420 | 833 | 1297 | 2606 | 559 | 60 |
| Patient-2 | 1131 | 1069 | 1079 | 1771 | 4161 | 193 | 30 |
| Patient-3 | 2404 | 3313 | 1650 | 2401 | 1653 | 1312 | 77 |
| Patient-4 | 3551 | 3518 | 1501 | 1621 | 2404 | 350 | 28 |

| **Supplementary Table 4.** snATAC-seq nuclei in each cell type | | | | | | | |
| --- | --- | --- | --- | --- | --- | --- | --- |
| Sample name | Inhibitory neuron | Excitatory neuron | OPCs | Microglia | Oligodendrocyte | Astrocyte | Endothelial cell |
| Control-1 | 1055 | 2452 | 342 | 653 | 2366 | 546 | 15 |
| Control-2 | 684 | 1329 | 181 | 366 | 1527 | 277 | 58 |
| Control-3 | 1255 | 2245 | 648 | 1148 | 1374 | 705 | 15 |
| Patient-1 | 1443 | 1746 | 484 | 971 | 1744 | 284 | 2 |
| Patient-2 | 937 | 930 | 758 | 1170 | 3033 | 351 | 13 |
| Patient-3 | 1205 | 2020 | 928 | 1111 | 916 | 597 | 26 |
| Patient-4 | 1845 | 1676 | 940 | 954 | 1402 | 211 | 31 |

| **Supplementary Table 5.** snRNA-seq nuclei in excitatory neuron subcluster | | | | | |
| --- | --- | --- | --- | --- | --- |
| Cluster | Controls | Patients |  |  |  |
| 0 | 2296 | 1782 |  |  |  |
| 1 | 1652 | 1863 |  |  |  |
| 2 | 2032 | 1466 |  |  |  |
| 3 | 1922 | 674 |  |  |  |
| 4 | 1003 | 1258 |  |  |  |
| 5 | 775 | 562 |  |  |  |
| 6 | 649 | 682 |  |  |  |
| 7 | 631 | 566 |  |  |  |
| 8 | 400 | 509 |  |  |  |
| 9 | 298 | 516 |  |  |  |
| 10 | 238 | 336 |  |  |  |
| 11 | 171 | 345 |  |  |  |
| 12 | 190 | 278 |  |  |  |
| 13 | 320 | 131 |  |  |  |
| 14 | 251 | 181 |  |  |  |
| 15 | 186 | 74 |  |  |  |
| 16 | 66 | 79 |  |  |  |
| 17 | 96 | 18 |  |  |  |

| **Supplementary Table 6.** snRNA-seq nuclei in OPCs subcluster | | | | |
| --- | --- | --- | --- | --- |
| Cluster | Controls | Patients |  |  |
| 0 | 880 | 1449 |  |  |
| 1 | 1105 | 1000 |  |  |
| 2 | 197 | 664 |  |  |
| 3 | 29 | 779 |  |  |
| 4 | 10 | 439 |  |  |
| 5 | 18 | 323 |  |  |
| 6 | 64 | 161 |  |  |
| 7 | 0 | 163 |  |  |
| 8 | 53 | 85 |  |  |
