# Supplementary material for: Excitatory neurons and oligodendrocyte precursor cells are vulnerable to focal cortical dysplasia type IIIa as suggested by single‐nucleus multiomics
Source: Clin Transl Med. 2024 Oct 23;14(10):e70072. doi: 10.1002/ctm2.70072 (PMC11497056; doi:10.1002/ctm2.70072)
Supplement: Supplementary file 3 — Supporting Information [file CTM2-14-e70072-s003.docx]

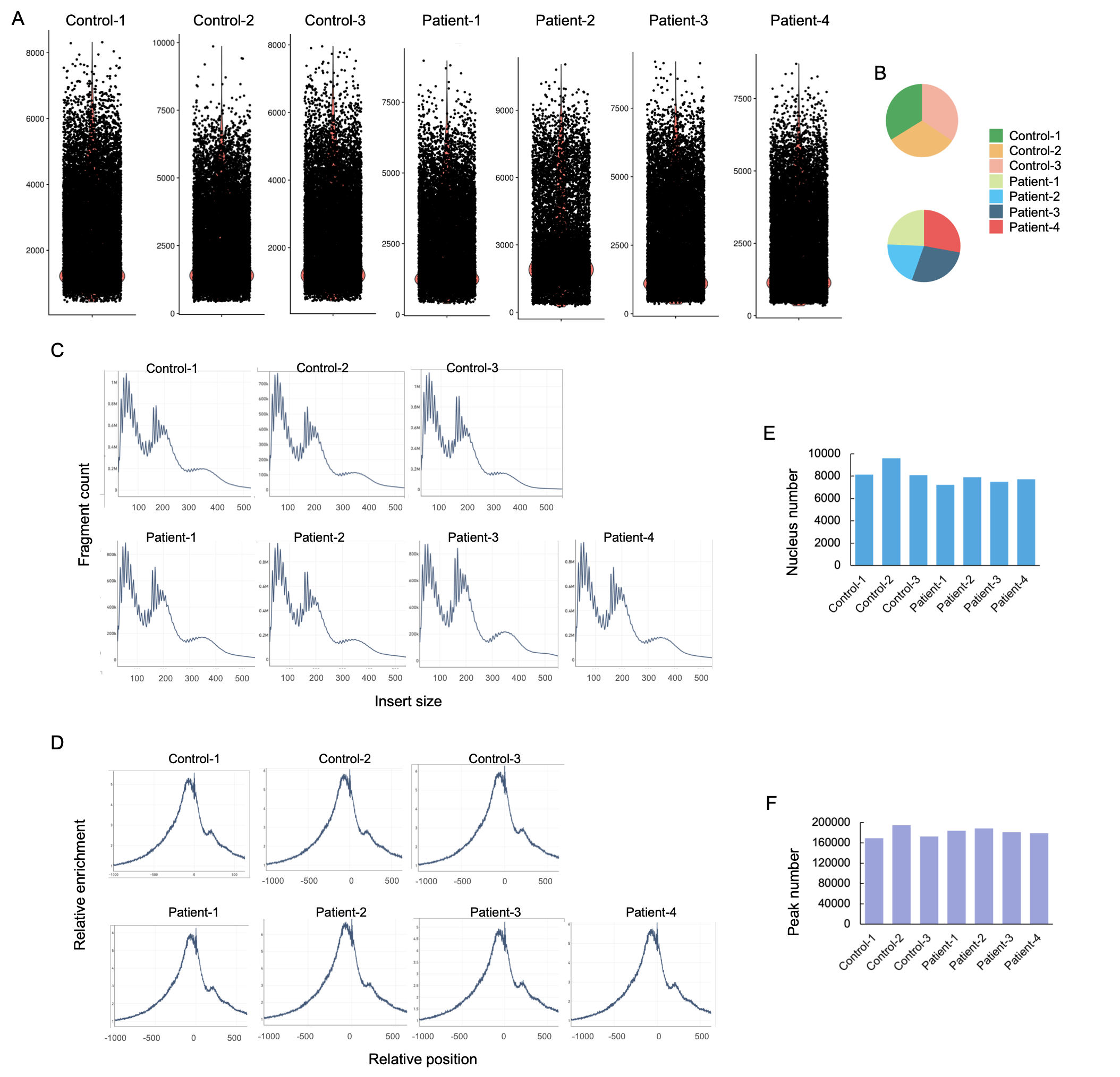


**Supplementary Fig. 2** Quality control metrics for snRNA-seq or snATAC-seq dataset. (A) The number of genes per cell of each sample in snRNA-seq data. (B) The pie charts showing the proportion of nuclei in each sample of the controls and patients is uniform and consistent. (C) The fragment size distribution of each sample in snATAC-seq data. (D) The enrichment of unique open chromatin fragment at TSS of each sample in snATAC-seq data. (E.F) The number of retained nuclei and peaks in each sample in snATAC-seq data.
