# Supplementary material for: Excitatory neurons and oligodendrocyte precursor cells are vulnerable to focal cortical dysplasia type IIIa as suggested by single‐nucleus multiomics
Source: Clin Transl Med. 2024 Oct 23;14(10):e70072. doi: 10.1002/ctm2.70072 (PMC11497056; doi:10.1002/ctm2.70072)
Supplement: Supplementary file 4 — Supporting Information [file CTM2-14-e70072-s007.docx]

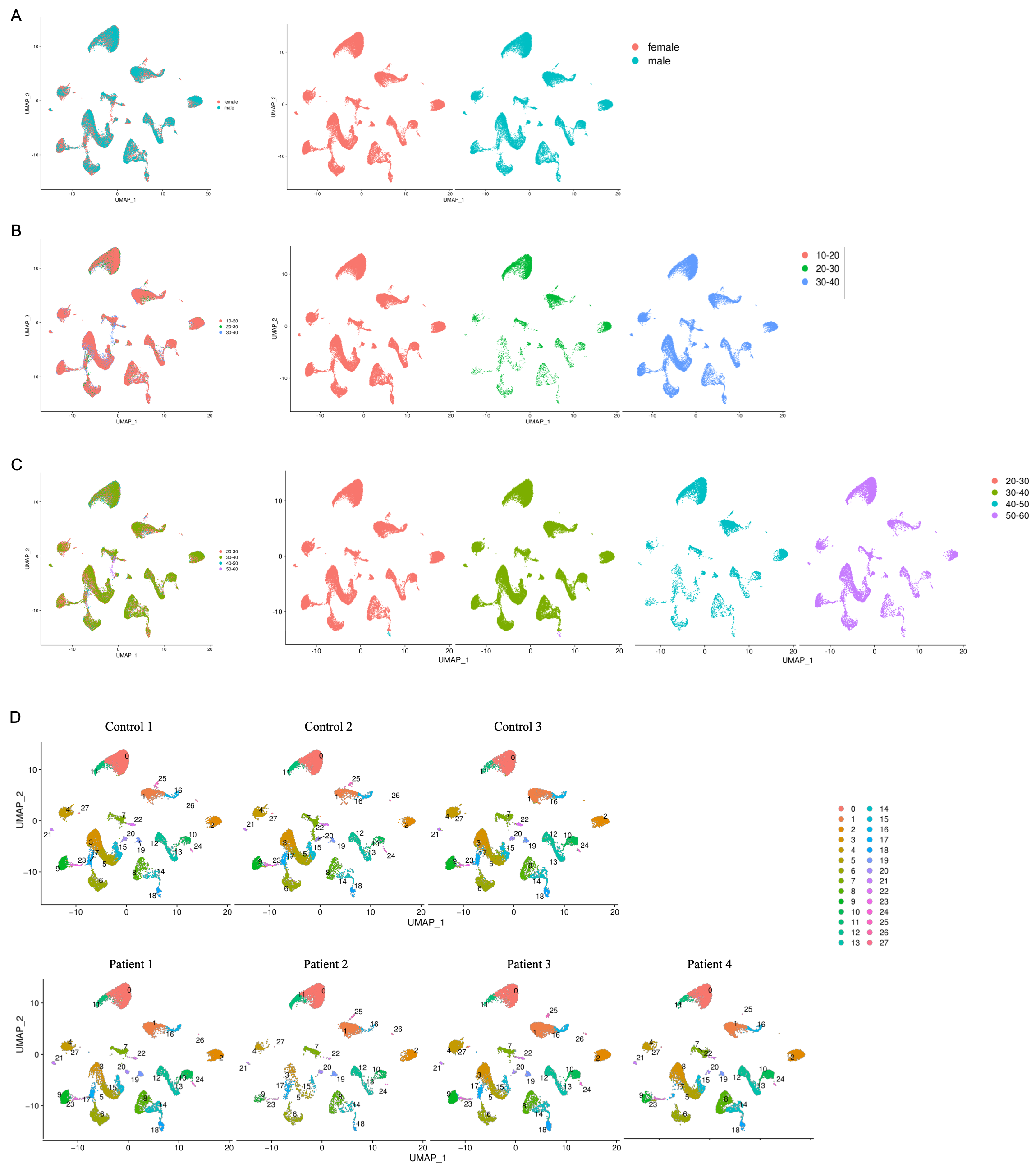
 **Supplementary Fig. 3** Categorical variables in snRNA-seq. (A) UMAP plots colored by sex (male, n=3, Patient-2，Patient-4 and Control-3; femal, n=4, Patient-1，Patient-3, Control-1 and Control-2). (B) UMAP plots colored by time from first onset to surgery (years): 10-20 years, n=4, Patient-1, Patient-3, Patient-4 and Control-1;20-30 years, n=1, Patient-2; 30-40 years, n=2, Control-2 and Control-3. (C) UMAP plots colored by age at surgery (years): 20-30 years, n=2, Patient-3 and Control-1; 30-40 years, n=3, Patient-1，Patient-4 and Control-3; 40-50 years, n=1, Patient-2; 50-60 years, n=1, Control-2. (D) UMAP plots of each sample.
