# Supplementary material for: Excitatory neurons and oligodendrocyte precursor cells are vulnerable to focal cortical dysplasia type IIIa as suggested by single‐nucleus multiomics
Source: Clin Transl Med. 2024 Oct 23;14(10):e70072. doi: 10.1002/ctm2.70072 (PMC11497056; doi:10.1002/ctm2.70072)
Supplement: Supplementary file 5 — Supporting Information [file CTM2-14-e70072-s005.docx]

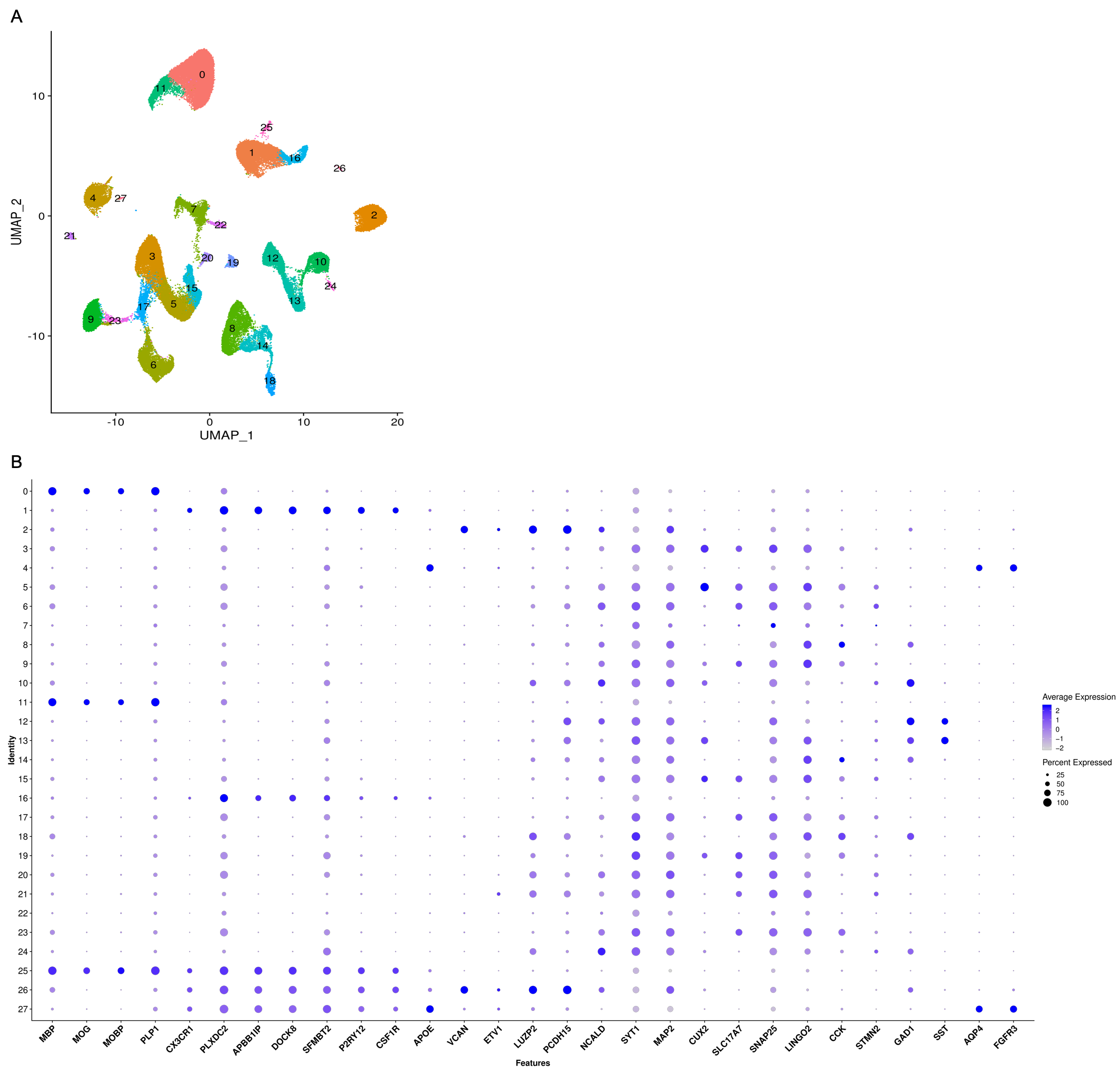
 **Supplementary Fig. 4** Gene expression patterns of specific markers in the snRNA-seq dataset. (A) UMAP visualization of the snRNA-seq dataset, including all clusters before the exclusion of cluster 7. (B) Dot plot illustrating gene expression patterns of subtype-specific markers in the snRNA-seq dataset. Rows and columns represent marker genes and subtypes, respectively. The color intensity represents the average expression level of this marker gene in a given subtype relative to the other subtypes. The size of the dot reflects the percentage of cells that express the indicated gene.
