# Supplementary material for: Excitatory neurons and oligodendrocyte precursor cells are vulnerable to focal cortical dysplasia type IIIa as suggested by single‐nucleus multiomics
Source: Clin Transl Med. 2024 Oct 23;14(10):e70072. doi: 10.1002/ctm2.70072 (PMC11497056; doi:10.1002/ctm2.70072)
Supplement: Supplementary file 6 — Supporting Information [file CTM2-14-e70072-s008.docx]

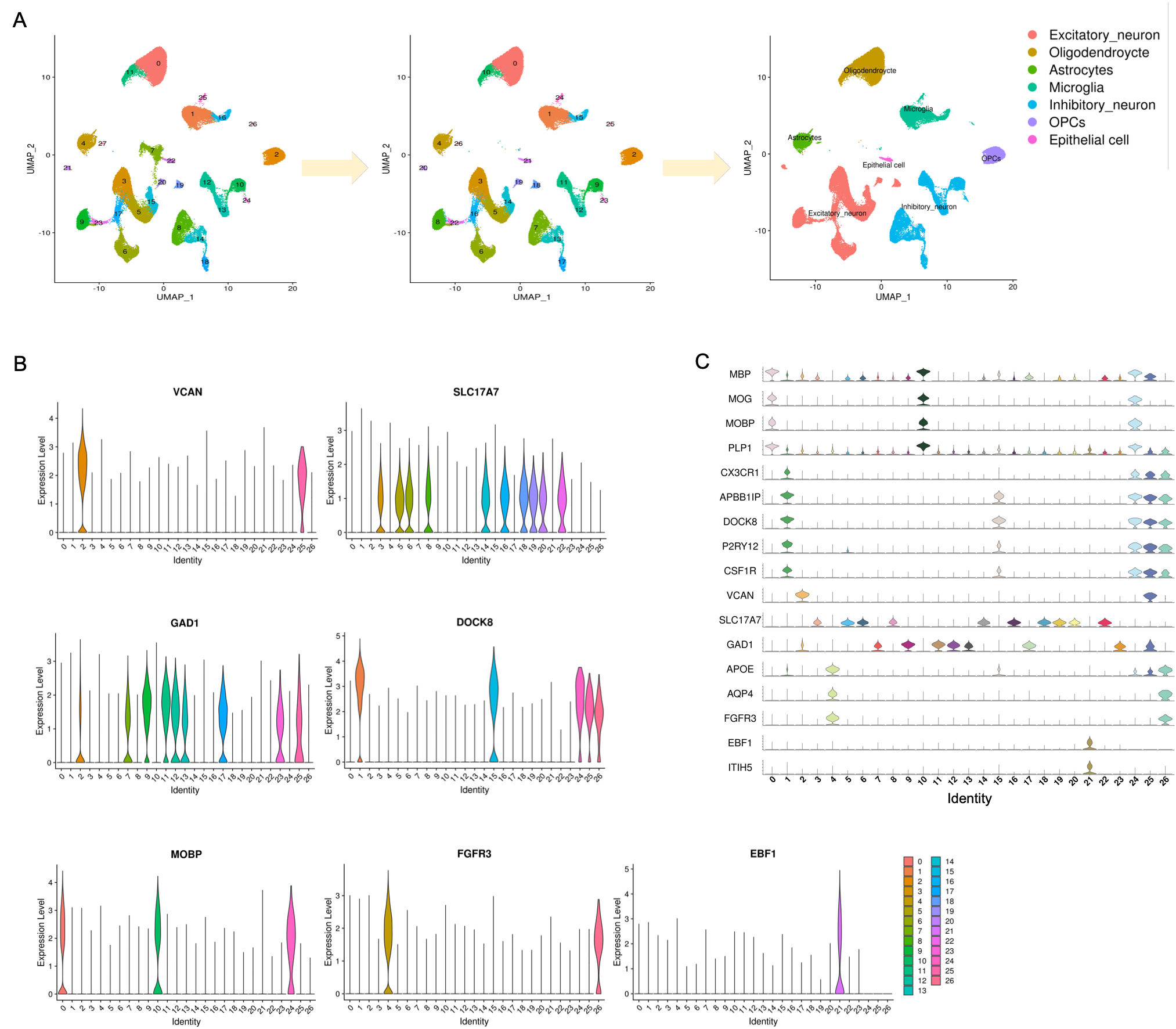
 **Supplementary Fig. 5** Cellular diversity on the human temporal neocortical. (A) UMAP representation of snRNA-seq dataset before and after removal of cluster 7, and coloured based on cluster assignment and cell-type. (B) Violin plots showing gene expression patterns of subtype-specific markers for principal cells in the snRNA-seq dataset. (C) Stacked violin diagram showing gene expression patterns of subtype-specific markers for principal cells in the snRNA-seq dataset.
