# Supplementary material for: Excitatory neurons and oligodendrocyte precursor cells are vulnerable to focal cortical dysplasia type IIIa as suggested by single‐nucleus multiomics
Source: Clin Transl Med. 2024 Oct 23;14(10):e70072. doi: 10.1002/ctm2.70072 (PMC11497056; doi:10.1002/ctm2.70072)
Supplement: Supplementary file 7 — Supporting Information [file CTM2-14-e70072-s002.docx]

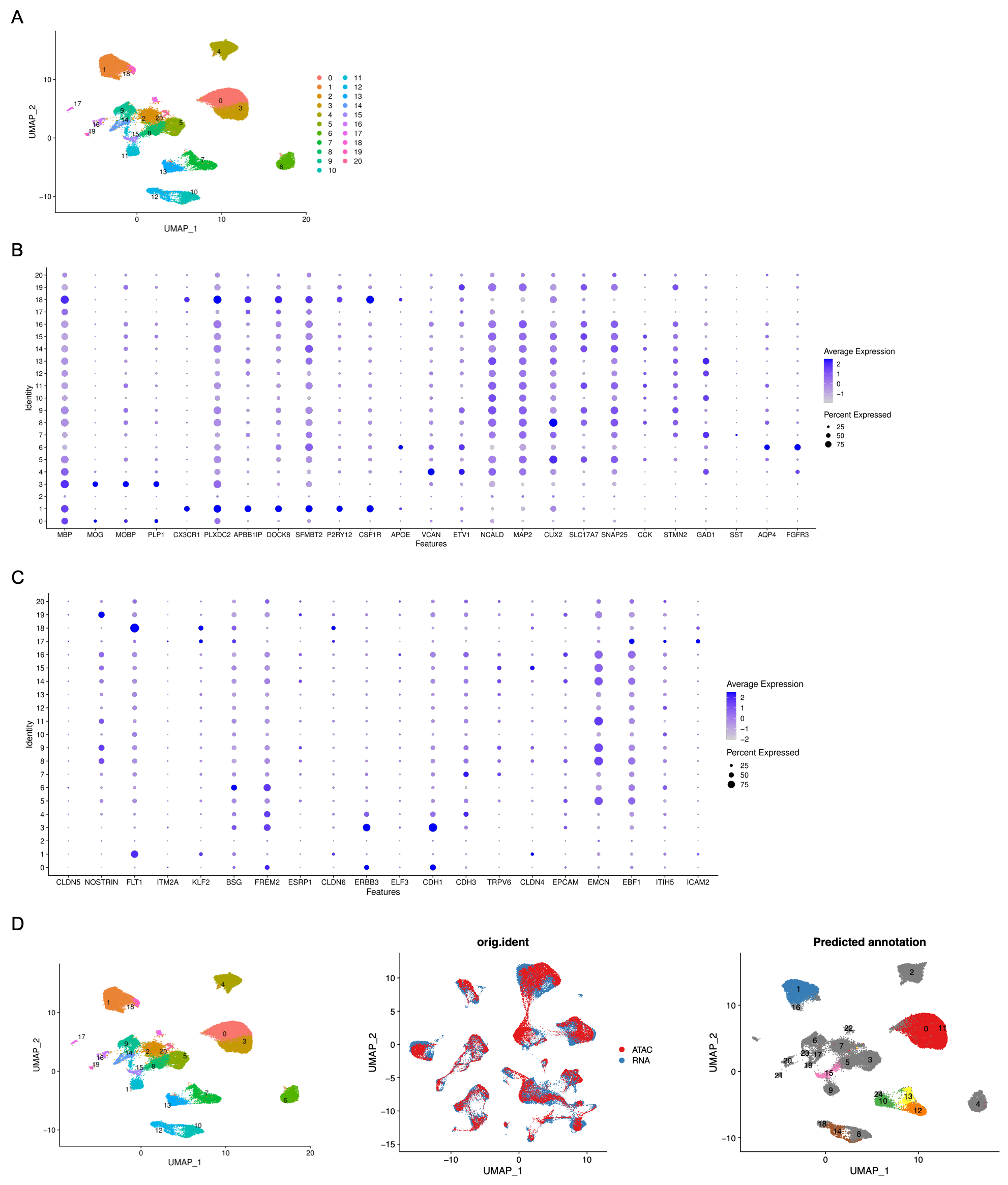
 **Supplementary Fig. 6** Gene activity patterns of specific markers in the snATAC-seq dataset. (A) UMAP representation of snATAC-seq dataset before removal of cluster 2. (B) Dot plot showing gene activity patterns of specific neuronal and glial markers in the snATAC-seq dataset. (C) Dot plot showing gene activity patterns of specific endothelial cell markers in the snATAC-seq dataset. Rows and columns represent marker genes and subtypes, respectively. (B.C) The intensity of the dot colour represents the average expression of the marker in a given subtype relative to the other subtypes. The size of the dot reflects the percentage of cells that express the indicated gene. (D) Multi-omics integration for disposing of the cluster 2 in snATAC-seq data. After integrating and transferring labels, the snATAC-seq data was assignmented by predicted cell-type. Cluster 2 in snATAC-seq dataset was predicted to correspond to cluster 7 in snRNA-seq dataset.
