# Supplementary material for: Excitatory neurons and oligodendrocyte precursor cells are vulnerable to focal cortical dysplasia type IIIa as suggested by single‐nucleus multiomics
Source: Clin Transl Med. 2024 Oct 23;14(10):e70072. doi: 10.1002/ctm2.70072 (PMC11497056; doi:10.1002/ctm2.70072)
Supplement: Supplementary file 8 — Supporting Information [file CTM2-14-e70072-s006.docx]

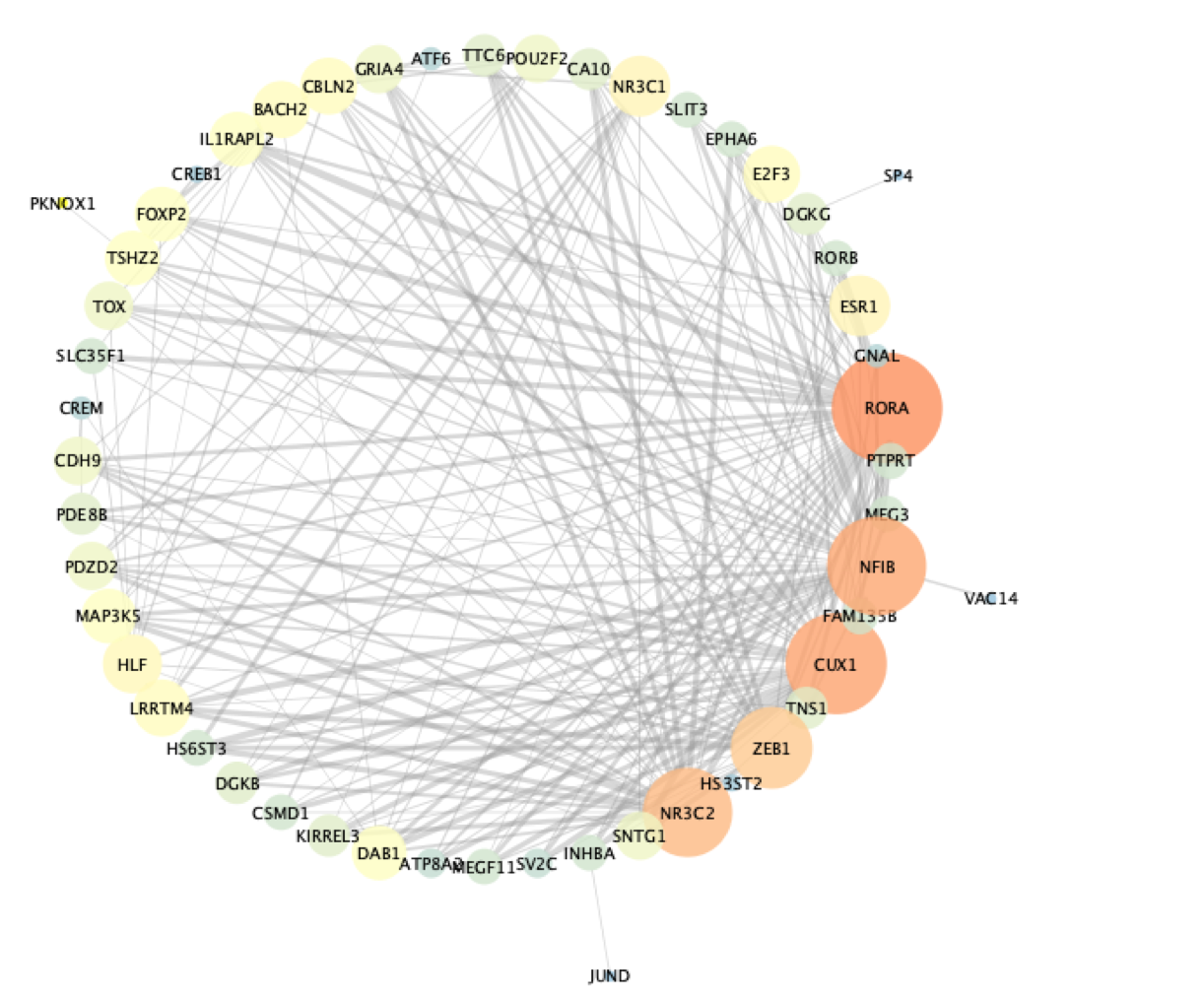


**Supplementary Fig. 7** TF regulatory networks showing the predicted candidate target hub genes for the TFs in ENs. The dot size indicates the intensity of regulation, and the larger the dot, the more genes and intensity regulated by the TF. The gray line indicates the existence of regulatory relationships.


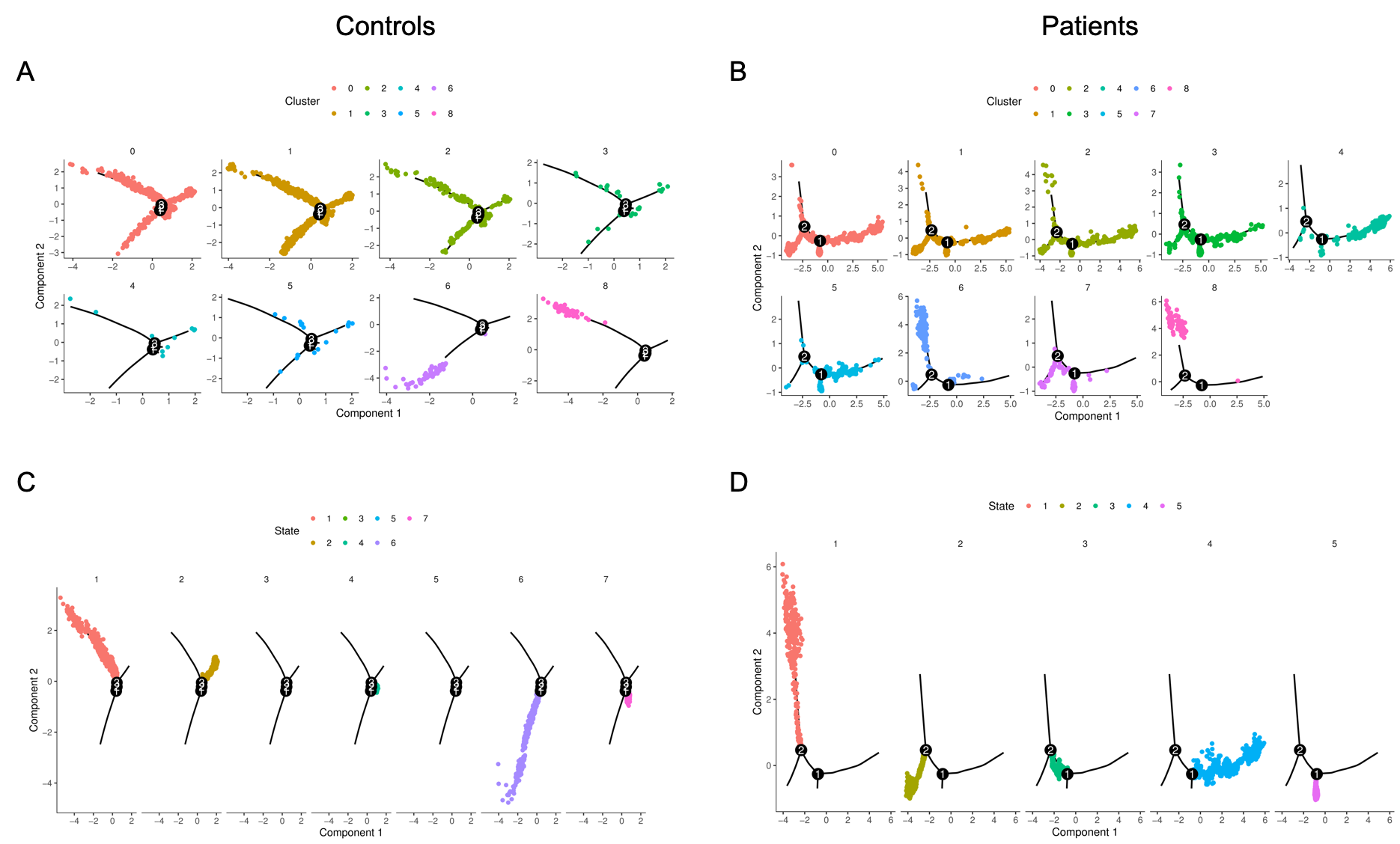


**Supplementary Fig. 8** Differentiation trajectories and state distribution of OPCs subpopulations. (A) Differentiation trajectorie of OPCs subpopulations in controls. (B) Differentiation trajectorie of OPCs subpopulations in FCD IIIa patients. (C) State distribution of OPCs subpopulations in controls. (D) State distribution of OPCs subpopulations in patients.
